# Mosquito  saliva enhances virus infection through sialokinin-dependent vascular leakage

**DOI:** 10.1073/pnas.2114309119

**Published:** 2022-06-08

**Authors:** Daniella A. Lefteri, Steven R. Bryden, Marieke Pingen, Sandra Terry, Ailish McCafferty, Emily F. Beswick, Georgi Georgiev, Marleen Van der Laan, Valeria Mastrullo, Paola Campagnolo, Robert M. Waterhouse, Margus Varjak, Andres Merits, Rennos Fragkoudis, Stephen Griffin, Kave Shams, Emilie Pondeville, Clive S. McKimmie

**Affiliations:** ^a^Virus Host Interaction Team, Leeds Institute of Medical Research, School of Medicine, Faculty of Medicine and Health, University of Leeds, Leeds, LS9 7TF, United Kingdom;; ^b^MRC-University of Glasgow Centre for Virus Research, Glasgow, G61 1QH, Scotland, United Kingdom;; ^c^Inflammatory Skin Disease Group, Leeds Institute of Rheumatic and Musculoskeletal Medicine, School of Medicine, Faculty of Medicine and Health, University of Leeds, Leeds, LS9 7TF, United Kingdom;; ^d^Section of Cardiovascular Sciences, Faculty of Health & Medical Sciences, University of Surrey, Guildford, GU2 7XH, United Kingdom;; ^e^Department of Ecology and Evolution, Swiss Institute of Bioinformatics, University of Lausanne, 1015 Lausanne, Switzerland;; ^f^Institute of Technology, University of Tartu, 50411 Tartu, Estonia;; ^g^School of Veterinary Medicine and Science, University of Nottingham, Sutton Bonington Campus, Nottingham, LE12 5RD, United Kingdom;; ^h^Leeds Institute of Medical Research, School of Medicine, Faculty of Medicine and Health, University of Leeds, Leeds, LS9 7TF, United Kingdom

**Keywords:** mosquitoes, arbovirus, inflammation, endothelium

## Abstract

Viruses transmitted by *Aedes* mosquitoes are an increasingly important global cause of disease. Defining common determinants of host susceptibility to this large group of heterogenous pathogens is key for informing the rational design of panviral medicines. Infection of the vertebrate host with these viruses is enhanced by mosquito saliva, a complex mixture of salivary-gland-derived factors and microbiota. We show that the enhancement of infection by saliva was dependent on vascular function and was independent of most antisaliva immune responses, including salivary microbiota. Instead, the *Aedes* gene product sialokinin mediated the enhancement of virus infection through a rapid reduction in endothelial barrier integrity. Sialokinin is unique within the insect world as having a vertebrate-like tachykinin sequence and is absent from *Anopheles mosquitoes*, which are incompetent for most arthropod-borne viruses, whose saliva was not proviral and did not induce similar vascular permeability. Therapeutic strategies targeting sialokinin have the potential to limit disease severity following infection with *Aedes*-mosquito-borne viruses.

## Significance

An increasingly important group of infectious agents is viruses spread by mosquitoes. When infected mosquitoes bite people, they inject virus and saliva into the skin. The saliva is biologically active and helps the mosquito feed but also helps the virus to infect and replicate. Sialokinin is a peptide in mosquito saliva that is unique within the insect world as having vertebrate-like tachykinin function, which can be proinflammatory. We show that sialokinin increases blood vessel permeability, allowing an influx of virus-permissive cells. Increasing the frequency of these cells in skin provides more opportunities for the virus to replicate. Inhibiting mosquito salivary factors holds promise as a panviral strategy for reducing disease burden caused by a wide variety of mosquito-borne viruses.

Mosquito-borne viruses are an important cause of debilitating and sometimes lethal infections. The most significant vectors are *Aedes* species mosquitoes that transmit several clinically important viruses (namely, arthropod-borne viruses [arboviruses]), including dengue (DENV), Zika (ZIKV), and chikungunya (CHIKV) viruses (1, 2). The recent emergence of arboviruses has led to explosive outbreaks of disease, for which there are no licensed medicines. Arboviruses are a large (>85 human pathogens), genetically diverse group of viruses that cause a wide spectrum of diseases (1, 3–5). This heterogeneity, combined with our inability to accurately predict the nature and timing of future epidemics, makes developing and stockpiling virus-specific medicines in a timely manner challenging. It is therefore important that we better understand common determinants of host susceptibility to infection and thereby inform the rational design of panviral medicines.

The extent of host susceptibility to arboviruses is defined by a combination of host, virus, and environmental factors. One such determinant is the host response to mosquito saliva that worsens outcomes to arbovirus infection (6–11). Infected mosquitoes transmit virus to the mammalian host as they probe the skin for blood and deposit saliva. Mosquito-derived factors enhance infection with many medically important viruses, including members of *Flavivirus*, e.g., DENV (8, 11–13) and West Nile virus (14–17); *Alphavirus*, e.g., Semliki Forest virus (SFV) (18) and CHIKV (19); and *Bunyavirales*, e.g., Rift Valley fever virus and Cache Valley virus (18, 20, 21). Enhancement of virus infection by saliva is apparent within hours, resulting in a higher quantity of virus in tissues and blood and more severe pathogenesis (6, 16, 22, 23). It is not clear how mosquito saliva increases host susceptibility to infection in such a rapid manner, although inflammatory responses that elicit an influx of virus-permissive monocytic cells are required (18). Despite evidence that some proinflammatory mosquito salivary factors can enhance ZIKV infection (24, 25), the mechanisms by which most salivary factors modulate vertebrate susceptibility to virus are still being defined (26, 27). Importantly, mosquito saliva is made up of a complex mixture of salivary-gland-gene–encoded products that, e.g., inhibit hemostatic processes, while salivary glands are also home to a diverse bacterial microbiota, the effects of which on virus infection remain mostly uncharacterized (28–30).

We show here that a remarkably rapid reduction in blood vessel barrier function, induced by *Aedes* saliva directly on endothelial cells, is necessary for enabling saliva enhancement of arbovirus infection. Importantly, we rule out a role for salivary bacterial microbiota and show that the factor involved was only proviral when applied in vivo and expressed specifically by female mosquitoes. *Anopheles* species mosquito saliva lacked the ability to enhance *Aedes*- or *Anopheles*-borne virus infection, and this correlated with an inability to induce vascular leakage. We exploited this naturally occurring difference to dissect the mechanistic basis by which *Aedes* saliva enhances infection with virus and to identify the requirement for the *Aedes* salivary factor sialokinin (SK). As such, we define an aspect of disease transmission that can inform the development of medicines with panviral potential that target SK.

## Results

### Mosquito Saliva Is Sufficient to Enhance Virus Infection and Worsen Clinical Outcome.

**Fig. 1.**
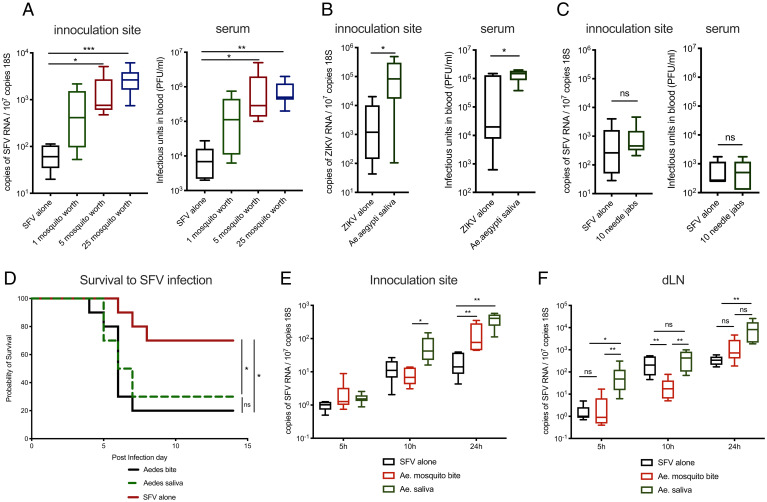
Mosquito saliva is sufficient to enhance virus infection and worsen clinical outcome. (*A*–*F*) Mice were inoculated with either 10^4^ PFU of SFV4 or 10^3^ PFU of ZIKV into skin of left foot (upper side), alone or following *Ae. aegypti* mosquito biting, or coinjected with *Aedes* saliva normalized for total protein content (mosquito salivated 0.3714 μg of protein on average). Viral RNA and host 18S were quantified by qPCR and viral titers of serum by plaque assays at 24 hpi. (*A*) Mice were injected with SFV alone or alongside saliva from 1, 5, or 25 mosquitoes (*n* = 6). (*B*) Mice were injected with ZIKV with or without saliva from five mosquitoes (1.86 μg of protein, *n* = 6). (*C*) Mice were infected with SFV following 1 or 10 repeated tissue piercings with a hyperfine needle. (*n* = 6). (*D*) Survival of mice infected with 10^4^ PFU of SFV4 (*n* = 10). (*E* and *F*) Mice were inoculated with SFV alone in resting skin, or into mosquito-bitten skin (5 bites), or into resting-skin mosquito saliva (1.86 μg of saliva protein, *n* = 8). *P < 0.05, **P < 0.01, ***P < 0.001, ns = not significant.

Mosquito bites involve skin tissue trauma and the deposition of saliva. Host responses to biting enhance incoming virus infection, as does the injection of experimentally derived homogenates of salivary gland tissue (6, 31). To define the factors in isolated saliva responsible for modulating host susceptibility to coinoculated virus in mice, we obtained saliva by forced salivation from adult *Aedes aegypti* female mosquitoes and coinjected this into skin with either the *Alphavirus* SFV or *Flavivirus* ZIKV (Fig. 1 *A* and *B* and *SI Appendix*, Fig. S1 *A* and *B*). Although the presence of just one mosquito’s salivation was sufficient to induce a 10-fold increase in virus by 24 h postinfection (hpi) (Fig. 1*A*), this was variable, likely as a consequence of salivation efficiency between mosquitoes (*SI Appendix*, Fig. S1*C*). Variation was much less pronounced when mice were coinjected with saliva obtained from 5 mosquitoes (Fig. 1 *A* and *B*). Repeated tissue piercing with our hyperfine needles in the absence of saliva, to simulate probing by mosquitoes, did not result in any virus enhancement (Fig. 1*C*). Coinoculation of virus with saliva also reduced survival to SFV to a similar extent as injection of the same titer virus into a mosquito bite (Fig. 1*D*). Increased quantities of virus RNA in skin were apparent from as early as 10 hpi, when virus was coinoculated with saliva, and additionally enhanced the early dissemination of virus to the draining lymph node (Fig. 1 *E* and *F*).

Host inflammatory responses to mosquito biting that recruit virus-permissive monocytic cells are important for modulating host susceptibility to virus (18). Here, we found that, similar to mosquito biting, saliva alone in the absence of virus induced the expression of proinflammatory genes *cxcl2*, *il1b*, and *ccl2*, while prototypic antiviral type I interferon (IFN)-stimulated genes (*isg15* and *ccl5*) whose expression correlates with host resistance to infection (32) were not altered (*SI Appendix*, Fig. S1*D*). In summary, we have optimized and defined our in vivo model for determining how saliva alone, in the absence of other mosquito bite or salivary gland tissue components, modulates host susceptibility to virus.

### Enhancement of Virus Infection In Vivo Requires Processes That Are Absent *Ex Vivo*.

To define whether mosquito saliva can directly modulate the susceptibility of the two principal cell types infected by SFV in the skin (fibroblasts and macrophages), we infected primary cultures of these cells in vitro in the presence or absence of saliva. However, the in vitro addition of mosquito saliva failed to recapitulate the virus-enhanced infection phenotype observed in vivo (*SI Appendix*, Fig. S2 *A*–*D*). This was a direct effect of saliva upon cells, rather than via direct action on virus, as cells pretreated with saliva and washed prior to infection (referred to here as saliva to cells) also exhibited increased resistance to infection (*SI Appendix*, Fig. S2 *B* and *D*). This in vitro virus infection inhibition by saliva was likely dependent on cellular responses activated by bacteria, as it was not observed with saliva from females treated with broad-spectrum antibiotics (*SI Appendix*, Fig. S2 *D*–*F*). *Aedes* salivary glands host a diverse microbiota (29) that may be secreted directly into saliva. However, it is not clear to what extent the mosquito salivary gland microbiota is secreted in vivo and whether it plays an important role in arboviral transmission.

In summary, these in vitro studies failed to recapitulate our in vivo phenotype in which virus infection is enhanced by saliva. To test whether crosstalk by skin cells or the presence of other cell types not present in the above cultures was required to mimic the in vivo phenotype, we assessed the susceptibility of intact skin explants to infection (32). However, these explants did not exhibit any increase in susceptibility to virus following exposure to saliva. This included the infection of explants derived from resting skin, mosquito-bitten skin, and explants derived from skin injected with saliva prior to biopsy (*SI Appendix*, Fig. S2 *G* and *H*). Together, this suggests that a key in-vivo–specific process was required for the saliva to enhance virus infection.

### A Heat-Sensitive Salivary Factor from Female Mosquitoes Enhances Virus Infection Independent of Bacterial Microbiota.

**Fig. 2.**
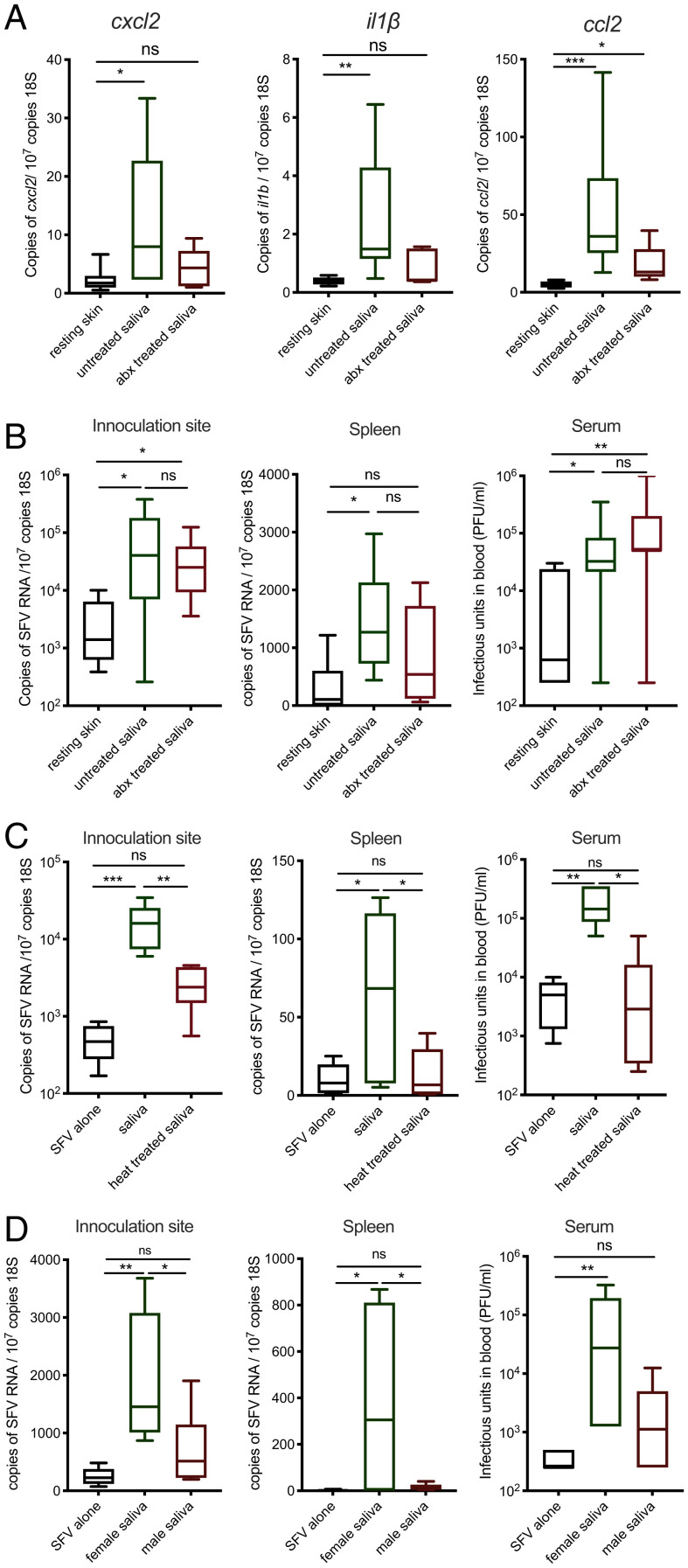
A heat-sensitive salivary factor from female mosquitoes enhances virus infection independent of bacterial microbiota. (*A*) Mouse skin was injected with 1.86 μg of saliva from control and antibiotic (Abx)-treated mosquitoes, and host expression of cxcl2, il1b, and ccl2 transcripts was assessed at 6 h (*n* = 6). (*B*–*D*) Mouse skin was inoculated with 10^4^ PFU of SFV4 alone or with *Ae. aegypti* saliva in the upper skin of the left foot. Viral RNA and host 18S were quantified from skin and spleen by qPCR and viral titers of serum by plaque assays at 24 hpi. (*B*) Female *Ae. aegypti* saliva from Abx-treated or untreated mosquitoes (*n* > 6). (*C*) Heat-treated (10 min at 95 °C) or untreated female *Ae. aegypti* saliva (*n* = 6). (*D*) Male or female *Ae. aegypti* saliva pooled from five mosquitoes combined, reared in the same cage (*n* = 6). *P < 0.05, **P < 0.01, ***P < 0.001, ns = not significant.

Next, we wanted to define which component in saliva was responsible for enhancing virus infection in vivo. Saliva from antibiotic-treated mosquito saliva was less able to upregulate the inflammatory chemokine *cxcl2* in vitro (*SI Appendix*, Fig. S2*I*). Because some inflammatory responses in vivo to mosquito biting can enhance infection with virus (18), we hypothesized that microbiota may account for the ability of saliva to enhance virus infection in vivo. However, while microbiota-depleted saliva induced lower quantities of inflammatory gene expression in skin (Fig. 2*A*), the microbiota was dispensable for the infection-promoting ability of saliva in vivo (Fig. 2*B*). Instead, the ability of saliva to enhance infection was sensitive to protein-denaturing temperatures (Fig. 2*C*), suggesting the proviral factor in saliva is likely a proteinaceous factor expressed and secreted by the mosquito salivary gland.

Many female-specific mosquito salivary components have evolved to facilitate blood feeding. In contrast, male mosquitoes that do not bite are naturally deficient in these blood-feeding factors (33). To determine whether the virus-enhancing salivary factor is one of these factors, we compared the ability of saliva from coreared male and females to modulate host susceptibility to virus. Crucially, female *Aedes* saliva enhanced virus infection to a significantly higher extent than male saliva. This was the case when either equal volumes of male/female saliva were used (Fig. 2*D*) or when saliva quantities were normalized for protein content, which was substantially lower in male saliva isolates (*SI Appendix*, Fig. S3*A*). Because blood feeding causes changes in gene expression within the salivary glands (34), we also assessed how saliva acquired from previously blood-fed and exclusively sugar-fed *Ae. aegypti* females modulated virus infection in mice. However, saliva from either group possessed similar virus-enhancing properties (*SI Appendix*, Fig. S3*B*). Thus, a female-specific salivary factor, which may have evolved to support efficient blood feeding, was responsible for enhancing the susceptibility to virus infection.

### *Anopheles* Mosquito Saliva Lacks the Ability to Enhance Virus Infection.

**Fig. 3.**
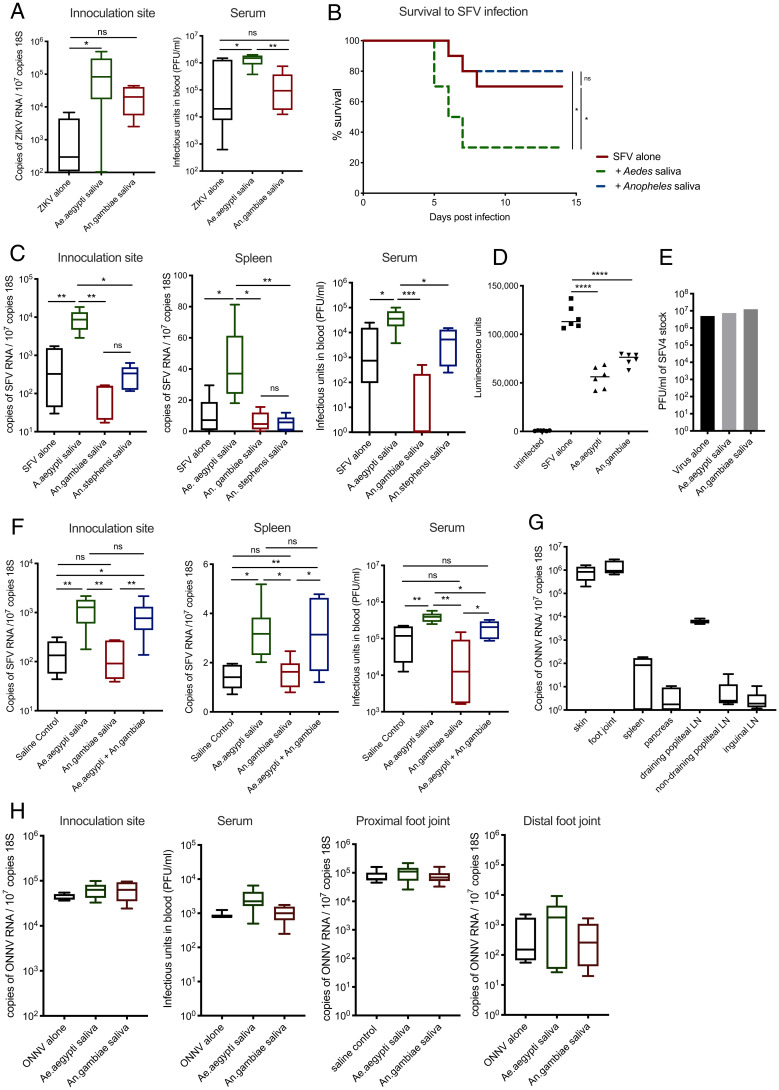
*Anopheles* mosquito saliva lacks the ability to enhance virus infection. (*A*–*C*) Mouse skin was inoculated with either 10^5^ PFU of ZIKV or 10^4^ PFU of SFV4 alone or with 1.86 μg of saliva of either *Ae. aegypti*, *An. gambiae*, or *An. stephensi*. Virus RNA and host 18S and serum viral titers were quantified at 24 hpi. (*B*) Survival of mice infected with 4 × 10^4^ PFU of SFV4. (*D*) Macrophages were infected with luciferase-expressing SFV at an multiplicity of infection of 0.1 alone or with 0.66 μg of protein of *Ae. aegypti, An. gambiae, or Ae. albpictus* saliva. Luciferase activity of tissue culture media was assayed at 6 hpi (*n* = 6). (*E*) BHK cells were infected with 10-fold serial dilutions of SFV4 ranging between 25,000 PFU and 0.25 PFU alone or with *Ae. aegypti* or *An. gambiae* saliva and then immediately overlayed with Avicel. PFUs were then assessed at 48 hpi. Shown here are representative PFUs for wells in which plaques were quantifiable. (*F*) Mouse skin was inoculated with 10^4^ PFU of SFV4 alone or alongside 1.86 μg of saliva of *Ae. aegypti, An. gambiae*, or both species. Virus RNA and host 18S were quantified from skin and spleen by qPCR and viral titers of serum by plaque assays at 24 hpi. (*G* and *H*) Mice were treated with 1.5 mg of anti-IFNAR antibodies (clone MAR1-5A3) and 24 h later were infected with 2 x10^5^ PFU ONNV s.c. in the skin (upper side of the left foot). (*G*) ONNV RNA quantities in tissues at 48 hpi were defined by qPCR to define tissue tropism. (*H*) Mouse skin was infected with ONNV alone or alongside 1.86 μg of saliva of either *Ae. aegypti* or *An. gambiae*. ONNV RNA and host 18S from tissues were quantified by qPCR, and serum viral titers were quantified via plaque assays at 48 hpi. *P < 0.05, **P < 0.01, ***P < 0.001, ****P < 0.0001, ns = not significant.

We next assessed whether saliva from other blood-feeding mosquito species has similar virus-infection–enhancing properties. It is not clear whether enhancement by saliva from mosquito species belonging to *Aedes* and *Culex* genera (*Culicinae* subfamily, *Culicidae* family), which are also important arbovirus vectors, is comparable in its ability to modulate vertebrate host susceptibility to virus. Neither is it clear if this is a general phenomenon elicited by all blood-feeding mosquito species saliva or whether saliva from arbovirus-vector–incompetent mosquitoes such as *Anopheles* (*Anophelinae* subfamily, *Culicidae* family) can also enhance infection. Because each mosquito genus has differing competence to transmit distinct viruses to different vertebrate species, we compared how saliva from each mosquito genus modulates vertebrate susceptibility to one reference virus (SFV) in one vertebrate host (mice). We found that *Aedes albopictus* and *Culex pipiens* saliva enhanced infection, with either SFV or ZIKV *in vivo*, to a similar extent as *Ae. aegypti* saliva (*SI Appendix*, Fig. S4 *A* and *B*). In comparison, *Anopheles* species mosquito saliva (*Anopheles gambiae* and *Anopheles stephensi)* could not (Fig. 3 *A*–*C* and *SI Appendix*, Fig. S4 *C*–*E*). Furthermore, unlike *Ae. aegypti*-bitten mice, *An. gambiae*–bitten mice exhibited similar host susceptibility to infection as resting skin (*SI Appendix*, Fig. S4*C*).

To exclude the possibility that *Anopheles* saliva may contain a unique factor that inhibited virus infectivity, we assessed whether saliva modulated virus infection in vitro. Similar to *Aedes* saliva, *Anopheles* saliva possessed some ability to protect cells from infection (Fig. 3*D*). However, in BHK-21 cells that lack IFN signaling, both species’ saliva had no effect on the ability of virus to replicate (Fig. 3*E*). In addition, we found that by mixing *Aedes* and *Anopheles* saliva together, we were able to restore the enhancement of virus infection in vivo (Fig. 3*F*). Together, this suggests that the ability to enhance virus infection is mosquito species specific and that the factor responsible in *Aedes* sp. saliva is missing in *Anopheles* saliva.

In comparison to *Aedes* mosquitoes, the widely distributed group of *Anopheles* species mosquitoes is not able to efficiently transmit arboviruses, despite them sharing an ecological niche that overlaps with arbovirus endemic areas (35, 36). The sole exception to this is the *Anopheles*-transmitted O'nyong'nyong virus (ONNV), whose genetic sequence is highly similar to CHIKV and similarly disseminates to joints (37, 38). While both *Aedes* and *Anopheles* have evolved salivary factors that facilitate efficient blood feeding (30), it is not clear why *Anopheles* sp. mosquitoes are such poor vectors of virus per se (37). It is conceivable that one reason is the absence of salivary factors that enhance the infection of virus in the mammals they feed on. To help explore this, we investigated whether *Anopheles* saliva, or indeed *Aedes* saliva, could modulate the infection of mice with ONNV, utilizing a newly developed ONNV mouse model in which virus efficiently replicated and disseminated from the skin inoculation site to joint tissue (Fig. 3*G*). Importantly, neither mosquito species saliva (Fig. 3*H*) or indeed the presence of a mosquito bite (*SI Appendix*, Fig. S4*F*) could modulate host susceptibility to infection with ONNV. Instead, ONNV was able to infect mice robustly, induce viremia, and disseminate to joint tissue without the need for saliva-based enhancement of infection, perhaps reflecting the absence of virus-infection–enhancing factor(s) present in its natural anopheline vector. Saliva from either species was also not able to modulate the ability of ONNV to infect and replicate in cultured cells (*SI Appendix*, Fig. S4*G*).

### Increased Vascular Permeability Induced by *Aedes* Saliva Enhances Virus Infection.

**Fig. 4.**
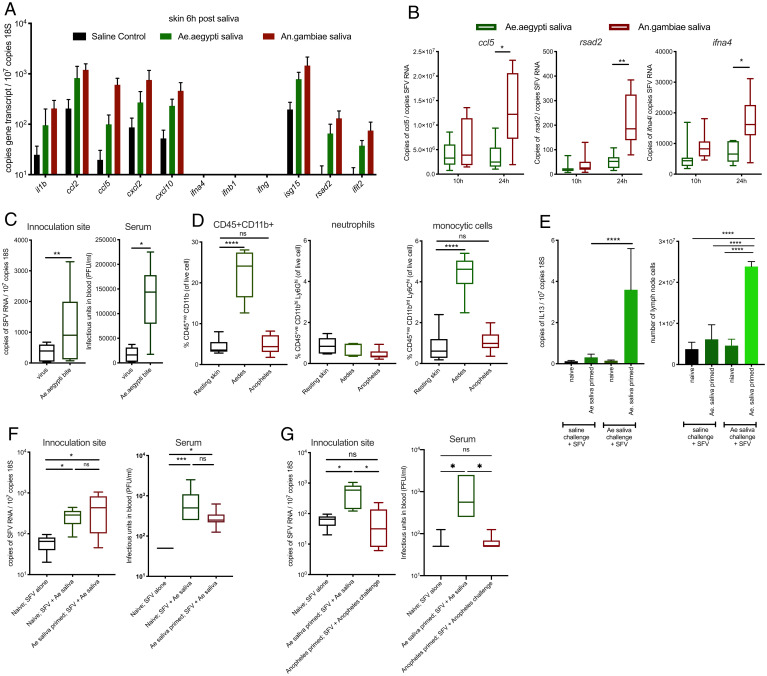
Immune sensing of *Aedes* saliva is not sufficient to enhance virus infection. (*A* and *B*) Mouse skin was injected with either saline control or 1.86 μg of saliva of either *Ae. aegypti* or *An. gambiae*. (*A*) Copy number of host transcripts in the skin was determined by qPCR at 6 h (*n* = 6). (*B*) Mice were inoculated with 10^4^ PFU of SFV4 with either *Ae. aegypti* or *An. gambiae* saliva and transcripts quantified by qPCR (*n* = 8). (*C*) Mice treated with IFNAR-1 blocking antibody a day prior to inoculation with 10,000 PFU SFV4 into mouse skin (resting or following mosquito biting). Viral RNA and host 18S were quantified from skin and spleen by qPCR and viral titers of serum by plaque assays at 24 hpi. (*n* = 6). (*D*) Mosquito-bitten mouse skin was injected with saliva from five mosquitoes of either *Ae. aegypti* or *An. gambiae*. At 2 h, skin from the inoculation site was biopsied and digested to release cells, and numbers of myeloid cells (CD45+CD11b+), neutrophils (CD45+CD11b+Ly6G+Ly6C^int^), and myelomonocytic cells (CD45+ CD11b+ Ly6G− Ly6C+ cells) were quantified (*n* = 6). (*E*–*G*) BALB/c mouse skin was inoculated with 10,000 PFU of SFV alone or with saliva. Mice were either naive to saliva or primed to saliva by prior injections of mosquito saliva weekly for four consecutive weeks. (*E*) IL-13 transcript expression and cell numbers of draining popliteal lymph nodes at 2 hpi. IL-13 transcripts were quantified by qPCR (*n* = 6). (*F*) Virus RNA in skin was measured by qPCR and serum virus quantified by plaque assay, at 24 hpi (*n* = 6) for mice primed with either *Aedes* saliva or saline alone. (*G*) Mice were presensitized to either *Aedes* or *Anopheles* saliva for 4 wk prior to SFV infection coinjected with respective species saliva. Expression of the viral SFV gene was measured using qPCR in the skin and serum virus quantified by plaque assay at 24 hpi. *P < 0.05, **P < 0.01, ***P < 0.001, ****P < 0.0001, ns = not significant.

*Aedes* mosquito biting induces the recruitment of monocytic cells, which can be infected by and replicate virus (6, 18). We hypothesized that *Anopheles* and *Aedes* saliva differ in their potency for activating key proinflammatory responses that attract monocytic cells. However, we found that the induction of most inflammatory responses to *Aedes* and *Anopheles* saliva was similar at the transcript level, including the key monocyte chemoattractant *ccl2*, suggesting that innate immune sensing for both was broadly analogous (Fig. 4*A*). *Anopheles* saliva more potently upregulated some IFN-stimulated genes, including *rsad2* and *ccl5* (Fig. 4 *A* and *B*). However, a more potent induction of antiviral IFNs by saliva did not account for its ability to modulate virus infection, as the enhancement of both SFV and ZIKV infection by *Aedes* saliva was IFN independent (Fig. 4*C*, for SFV; Fig. 3*A* and *SI Appendix*, Fig. S4*C*, for ZIKV). Importantly, we found that despite similar *ccl2* chemokine expression, *Aedes* saliva resulted in a robust and rapid influx of monocytic cells by 2 h, while *Anopheles* saliva did not (Fig. 4*D*).

Heightened skin inflammatory responses to saliva can also occur in those previously exposed to biting mosquitoes. However, we found that the vaccination of mice with saliva prior to infection did not result in any further modulation of host susceptibility to virus. Here, mice were immune sensitized to saliva or saline control by four weekly injections and on week five were injected with either SFV alone or SFV with saliva. At 2 hpi, saliva-primed mice exhibited significantly elevated draining lymph node cellularity and IL-5 and IL-13 expression and increased serum IgE indicative of immune sensitivity to saliva (Fig. 4*E* and *SI Appendix*, Fig. S5 *A* and *B*). However, *Aedes*-saliva–primed mice showed no difference in their susceptibility to SFV when compared to saliva-naïve mice (Fig. 4*F*). *Anopheles*-saliva–primed mice also exhibited no difference in their susceptibility to virus, such that they were similar to saliva-naive mice injected with virus alone (Fig. 4*G*). Thus, immune sensing of mosquito saliva alone is not sufficient to enhance virus infection.

**Fig. 5.**
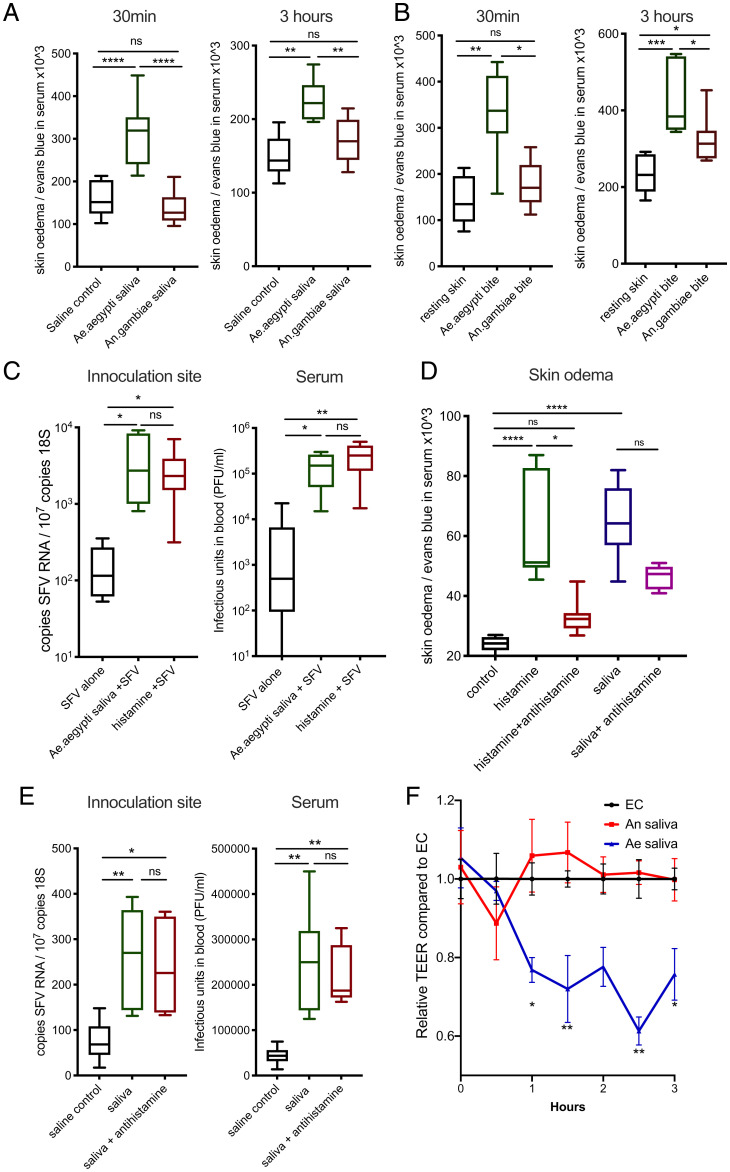
Increased vascular permeability induced by *Aedes* saliva enhances virus infection. (*A* and *B*) Mice administered i.p. with Evans blue were injected with 1.86 μg of mosquito saliva in the skin or exposed to up to three bites from *Ae. aegypti* or *An. Gambiae*. The extent of edema was assessed by quantification of Evan’s blue dye leakage into skin at 30 min and 3 h postsaliva/biting via colorimetric assay (*n* = 6). (*C*) Mouse skin was inoculated with 10^4^ PFU of SFV4 alone or with either *Ae. aegypti* saliva or 10 μg of histamine dihydrochloride. SFV RNA and host 18S and serum viral titers were quantified at 24 hpi. (*D*) Mice were administered i.p with Evans blue and then given either control or antihistamines s.c. (0.5 mg cetirizine in 100 μL, 0.02 mg loratadine in 100 μL, and 0.1 mg of fexofenadine in 200 μL) 1 h prior to saliva injection. Mouse skin was then injected with saliva from five *Ae. aegypti*, 10 μg of histamine dihydrochloride, or *An. gambiae* saliva, or a combination of *An. gambiae* saliva and 10 μg of histamine. Quantity of skin Evans blue was measured after 30 min by colorimetric assay. (*E*) Mice were pretreated with either control saline or antihistamines s.c. (0.5 mg cetirizine in 100 μL, 0.02 mg loratadine in 100 μL, and 0.1 mg of fexofenadine in 200 μL) 1 h prior to infection and then skin inoculated with 10^4^ PFU of SFV4 alone or with *Ae. aegypti* saliva. SFV RNA and host 18S and serum viral titers were quantified at 24 hpi. (*F*) Human primary endothelial cell monolayers were treated with either control saline or *Ae. aegypti* or *An. gambiae* saliva; electrical resistance across the monolayer was assessed longitudinally. *P < 0.05, **P < 0.01, ***P < 0.001, ****P < 0.0001, ns = not significant.

During our experiments above, we had anecdotally noted that *Anopheles* saliva, unlike *Aedes* saliva (11), did not cause a noticeable amount of fluid to accumulate at the site of inoculation. Components of *Aedes* saliva are known in an experimental setting to vasodilate constricted aortic ring tissue (39), although its effect on dermal postcapillary venules (through which most leukocyte entry occurs) is not described. We hypothesized that *Aedes* saliva may lead to an unusually rapid influx of monocytic cells (Fig. 4*D*) through its ability to induce skin vasculature barrier leakage directly and thereby underpin its ability to enhance virus infection. We therefore quantified levels of edema as a measure for vasculature barrier leakage following injection with saliva or exposure to mosquito biting; both *Ae. aegypti* saliva and bites led to significantly more edema than that by *An. gambiae* (Fig. 5 *A* and *B* and *SI Appendix*, Fig. S6 *A* and *B*).

We next asked whether the induction of vascular leakage was immune mediated or due to direct action of salivary factors on the vasculature. Histamine is the principal host factor that mediates early/immediate edema in response to exogenous agents, infection, or injury (40), and its injection into skin made mice more susceptible to SFV (Fig. 5*C*), suggesting that endothelial leakage alone is sufficient to promote virus infection. However, we found that histamine was not necessary for host responses to saliva, as treatment with antihistamines did not significantly suppress saliva-induced edema (although control histamine-induced edema was suppressed) or virus enhancement by saliva (Fig. 5 *D* and *E*). This agrees with studies in humans, in which prior histamine administration had little effect on skin inflammatory responses to *Aedes* mosquito biting (41). Together with our findings above (Fig. 4*F*) that immune hypersensitization to saliva did not modulate host susceptibility to virus, we concluded that a factor in *Aedes* saliva acts directly on blood vasculature to induce barrier leakage, in an immune-response–independent manner. Therefore, we decided to assess whether endothelial cell barrier function was directly modulated by saliva and if this was mosquito species specific. Here, a monolayer of primary blood endothelial cells was allowed to reach confluence and establish a barrier, as assessed by electrical resistance. Monolayers were then treated with either control saline or *Ae. aegypti* or *An. gambiae* saliva, and electrical resistance across the monolayer was assessed longitudinally. Importantly, cultured endothelial cell monolayers responded to *Aedes* saliva, but not *Anopheles* saliva, by rapidly decreasing endothelial barrier function (Fig. 5*F*).

Collectively, by comparing host responses to *Aedes* saliva and *Anopheles* saliva, we have identified blood vascular leakage as a key feature associated with female *Aedes* saliva enhancement of virus, a phenotype that is recapitulated with histamine-induced barrier loss, but which is independent of innate and adaptive immune sensing of saliva. Instead, we found that a salivary factor in *Aedes* saliva was responsible for directly inducing endothelial barrier loss.

### *Aedes* SK Is Sufficient to Induce Blood Vasculature Barrier Leakage and Enhance Virus Infection.

The saliva of blood-feeding arthropods contains many factors that facilitate blood feeding, putatively via altered vascular function. In *Ae. aegypti*, one such hypothesized factor is SK, a 1,400-Da peptide product of the pro-SK precursor–encoding gene (AAEL000229). SK belongs to the family of tachykinin‐like peptides (39, 42) and is expressed in the female salivary gland (43). We sought to define the evolutionary conservation of homologs in other blood-feeding insects and determine whether SK has any role in modulating mammalian vascular barrier function and thereby host susceptibility to virus infection.

**Fig. 6.**
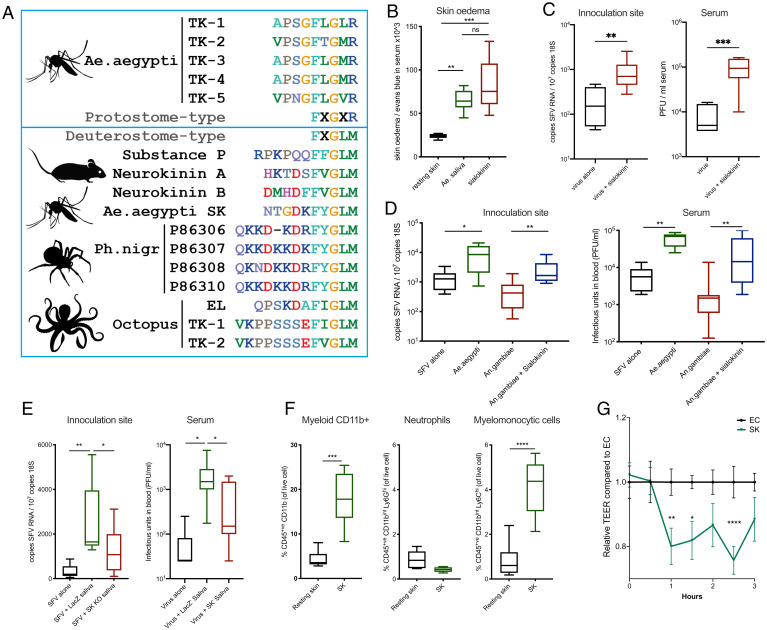
*Aedes* SK is sufficient to induce blood vasculature barrier leakage and enhance virus infection. (*A*) Protostome- and deuterostome-type TRPs. The *Aedes aegypti Tachykinin* (AAEL006644) gene encodes five TRPs (*Ae. aegypti* TK1–5) that match the protostome-type FXGXRamide signature (*Top* box). In contrast, the SK tachykinin-like peptide (*Ae. aegypti* SK, 1,400 Da) matches the deuterostome-type FXGLMamide signature (*Bottom* box). This motif is similar to mammalian tachykinins Substance P and Neurokinins A and B. Rare examples of other protostome species with peptides matching the deuterostome-type signature include octopuses (EL, eledoisin; TK, tachykinin) and a venomous spider (Ph.nigr). (*B*) Mice were injected i.p. with Evans blue, and 1 h later, skin was injected with either 1.86 μg of *Ae. aegypti* saliva or 1 μg of SK. Skin samples were collected 30 min post injection. (*C*–*E*) Mouse skin was inoculated with 10^4^ PFU of SFV4 alone or with saliva from either *Ae. aegypti* or *An. gambiae*, with or without supplementation with 1 μg SK peptide. SFV RNA and host 18S were quantified by qPCR and serum viral titers quantified by plaque assay at 24 hpi. (*E*) Virus was administered alone or alongside saliva from *Ae. aegypti* females injected with dsRNA LacZ or dsRNA SK. (*F*) Resting mouse skin was injected with SK alone. At 2 h, skin from the inoculation site was biopsied and digested to release cells, and numbers of myeloid cells (CD45+CD11b+), neutrophils (CD45+CD11b+Ly6G+Ly6C^int^), and monocytic cells (CD45+ CD11b+ Ly6G− Ly6C+) were quantified (*n* = 6). (*G*) Human primary endothelial cell monolayers were treated with either control saline or 1 μM SK alone, and electrical resistance across the endothelial cell (EC) monolayer was assessed longitudinally. *P < 0.05, **P < 0.01, ***P < 0.001, ****P < 0.0001, ns = not significant.

Tachykinin‐related peptides (TRPs) have been identified from many insect species (44), and in *Ae. aegypti*, five TRPs are encoded by Tachykinin (AAEL006644), a gene with single-copy orthologs across protostome invertebrates (45, 46). Intriguingly, we found that the SK tachykinin-like peptide (NTGDKFYGLMamide) more closely resembles typical deuterostome-type (FXGLMamide) than protostome-type (FXGXRamide) peptides (Fig. 6*A*). In addition, SK is also recognized by the PROSITE tachykinin family signature pattern (PS00267), which matches deuterostome-type but not protostome-type peptides. Together, this suggests that SK might primarily target exogenous vertebrate receptors rather than endogenous mosquito receptors. Indeed, SK elicits intestinal contractions in mammals and cross-reacts to antibodies that recognize the prototypic mammalian tachykinin substance P (39, 42). Substance P is a neuropeptide that is expressed widely in the central nervous system and is best known for its ability to activate nausea, although roles in mediating neurogenic inflammation and itch responses in the periphery have also been described (47–49). Crucially, the presence of such deuterostome-type tachykinin-like peptides in arthropods appears to be extremely rare, with the exception of four peptides from the highly venomous Brazilian wandering spider, whose venom induces edema in mammals (50). Beyond arthropods, matching peptides were identified in three octopus species, where vasoactive effects might be important for immobilizing prey.

We also examined the available insect species genomics data, to define which specifies have SK-like genes. No homologs were identified from the gene family data at VectorBase (51) or at OrthoDB (52). Sequence searches of the National Center for Biotechnology Information (NCBI) nonredundant protein and nucleotide databases returned hits only to the *Ae. aegypti* pro-SK precursor itself. We also searched the newly available genome assembly for *Culex tarsalis* (53) and all nucleotide data at VectorBase (25 mosquito species, genomes, transcriptomes, complementary DNAs [cDNAs], and expressed sequence tags [ESTs]), but only recovered the *Ae. aegypti* pro-SK precursor. The large sampling of *Anopheles* species, including several high-quality chromosome-level assemblies, lends confidence to the conclusion that this gene is not present in anophelines. The lack of hits in *Ae. albopictus* and two *Culex* species suggests that it could be specific to *Ae. aegypti.* However, the gene is located in a repeat-rich gene desert (nearest neighbors are 0.48 Mbp and 0.99 Mbp) at the start of *Ae. aegypti* chromosome one. Such repeat-rich regions can be challenging to sequence and assemble; therefore, it remains possible that other culicine mosquitoes do possess pro-SK precursor–encoding genes.

We hypothesized that SK may account for the ability of *Aedes* saliva to promote vascular leak and thereby enhance virus infection. Indeed, when coinoculated with virus in the absence of saliva, synthesized SK caused a rapid induction of edema and enhanced virus infection (Fig. 6 *B* and *C*). When SK was added to *Anopheles* saliva, viral enhancement was successfully generated (Fig. 6*D*). However, because we had found that the *Aedes* saliva enhancement of virus infection was partially heat sensitive to treatment at 95 °C for 10 min (Fig. 2*C*), and because previous work has shown some SK function was resistant to heat treatment for 2 min (39), we wanted to assess whether our synthesized SK was likewise heat sensitive. In agreement with both studies, we found that while a 2-min heat treatment did not significantly decrease the ability of SK to induce edema in vivo, a 10-min treatment was sufficient to significantly decrease SK-peptide–induced edema (*SI Appendix*, Fig. S7*B*).

Enhancement of virus by SK was dose dependent, as a 50-fold lower dose of SK caused only a modest increase in host susceptibility to virus (*SI Appendix*, Fig. S7*A*), suggesting that other components within saliva may prime host responses to SK and thereby modulate host susceptibility to virus. Therefore, to determine whether SK expressed by *Aedes* saliva gland was necessary to enhance virus infection, we silenced the SK gene in adult female *Ae. aegypti* mosquitoes (*SI Appendix*, Fig. S7*C*), by injection of a double-stranded RNA (dsRNA) targeting the SK gene. Saliva derived from these mosquitoes lost much of its ability to enhance infection compared to dsLacZ-dsRNA–injected control females (Fig. 6*E*). The extent of knockdown of SK gene expression (sevenfold reduction of median; *SI Appendix*, Fig. S7*C*) was similar to the magnitude of reduction in virus serum titers by 24 hpi (10-fold reduction, Fig. 6*E*). The reduction in skin virus RNA quantities in SK-deficient-saliva–injected mice was more modest (1.5-fold reduction, Fig. 6*E*). Importantly, similar to whole *Aedes* saliva, the ability of SK alone to enhance virus infection correlated with its ability to selectively and rapidly recruit monocytic cells in vivo (Fig. 6*F*). This effect was via direct action on endothelial cells, as an exposure of monolayers to SK resulted in rapid loss in electrical resistance and thereby an increase in permeability (Fig. 6*G*), similar in kinetics and magnitude as that observed with *Ae. aegypti* saliva (Fig. 5*F*). Thus, we have identified a factor in *Aedes* saliva, SK, which induces blood vascular barrier permeability, leading to edema and a rapid influx of virus-permissive monocytes that enhances infection with virus.

## Discussion

In this report, we have described how a mosquito saliva component is required for the successful establishment of virus infection in the mammalian host. The transmission of virus from mosquito to vertebrate skin constitutes an important bottleneck that is a critical step for viruses to overcome. Factors that provide a replicative advantage for viruses at this key stage of infection are key for defining overall outcome. This includes components within mosquito saliva that trigger an influx of virus-permissive monocytic cells that efficiently replicate virus. Salivary proteases have also been shown to modulate host susceptibly to virus (8).

Here, we show that the host response to *Aedes* mosquito saliva is remarkably rapid, inducing peak edema and monocyte influx within minutes postexposure. Surprisingly, early immune sensing, as shown by extent of innate immune cytokine expression and adaptive immune hypersensitivity responses to saliva, was not required for vascular permeability and thereby enhancement of virus infection. Instead, we found that early proviral vascular leakage occurred through a direct pharmacological effect induced by a mosquito-encoded substance P mimic, SK, that has likely evolved to facilitate optimal blood feeding. Subsequent immune responses that follow early saliva-induced leukocyte entry likely act to further extend skin residency time and tissue micropositioning of leukocytes, e.g., to sites of virus replication, and thereby increase the probability of their infection (18). Future studies that extend these findings to human responses would be valuable.

Our observation that early enhancement of infection by saliva was only evident in vivo is consistent with the requirement for a circulatory system that was absent in our in vitro and ex vivo models. The lack of infection enhancement in vitro was initially surprising, as several previous studies have demonstrated an enhancing effect of mosquito salivary gland extracts in cultured cells with other arboviruses. Together, these studies have utilized a number of distinct cell lines and virus strains (54). It is not clear why we could not replicate these findings with our models. Putatively, the use of salivary gland tissue homogenates in these studies, rather than mosquito saliva itself, may explain the difference in findings, as could the use of virus. Our data also demonstrate the limitation of exclusively using in vitro models that do not fully replicate all features of virus infection during arthropod biting.

Importantly, we have excluded a role for other salivary components that have been previously hypothesized to modulate vertebrate susceptibility to infection. This includes bacterial microbiota, which although we found did augment some skin inflammatory responses, did not modulate outcome to virus infection. Instead, we identified a role for a female-salivary-gland–specific factor. The ability of female *Aedes* saliva, but not *Anopheles* saliva, to act directly on blood endothelial cells to promote barrier loss, rather than solely through immune sensing, enabled a rational approach in selecting SK as a candidate to study. As such, we showed that SK was sufficient, and necessary, to induce endothelial barrier permeability and enhance virus infection. In addition, our evolutionary studies have shown that the existence of vertebrate-like immune neuropeptides, such as SK, that mimic substance P within the arthropods, is exceptionally uncommon and appears to be unique to the *Aedes* mosquitoes.

The observation that *Anopheles* saliva does not enhance virus is also key for considering its implication for understanding vectorial capacity for arbovirus transmission. Our data suggest that coevolution of arboviruses in competent vectors, such as *Aedes*, has led to the virus utilizing vector-specific factors to increase their infectiousness. We suggest this will modulate the overall effectiveness of arbovirus transmission by the competent vector, leading to an increase in vectorial capacity and thereby a high disease burden.

Together, our studies have defined an important determinant of the clinical outcome to infection at the arthropod/mammalian interface. We suggest this can inform the development of strategies that target SK to reduce host susceptibility to globally important arboviral diseases. This could include vaccines that target mosquito salivary factors (24, 25), although any such an approach must be done carefully to avoid off-target effects, which we suggest might be best achieved by targeting the N terminus of soluble SK, which lacks homology to human tachykinins. Vaccines would also need to be carefully designed to elicit protective Th1-associated IgG2 antibodies that lack the immunopathology associated with hypersensitivity reactions mediated by Th2-associated IgG1 and IgE responses. In conclusion, therapies that target mosquito saliva may have wide applicability, as mosquito saliva enhancement is widely observed for these viruses.

## Materials and Methods

### Mosquito Strains.

The following mosquito strains were used: *Ae. aegypti* (Liverpool strain), *Ae. albopictus* (La Providence strain), *Cx. pipiens* (slab strain), *An. gambiae* (Kisumu strain), and *An. stephensi* (SDA-500 strain).

### Virus Strains.

SFV4 and SFV6 stocks were generated from plasmids containing corresponding infectious cDNA (icDNA) sequences (55). SFV6-2SG-GLuc (Gaussia luciferase) is a modified SFV6 where a sequence encoding Gluc marker is inserted under a duplicated sub-genomic (SG) promoter positioned at a 3′ direction of the structural reading frame. ONNV-2SG-ZsGreen represents a modified ONNV of Chad isolate where a sequence encoding for ZsGreen is inserted between native and duplicated SG promoters of ONNV. Plasmids containing the icDNAs of SFV4, SFV6, or SFV-6-2SG-GLuc were electroporated into BHK-21 cells to generate infectious virus with two pulses at 250V for 0.8 s. A plasmid to rescue ONNV-2SG-ZsGreen was linearized first with PmeI (New England BioLabs) to prepare it for run-off transcription; RNA was transcribed using a MEGAscript SP6 transcription Kit (Thermo Fisher Scientific) in the presence of Ribo m7G Cap Analog (Promega). The RNA was transfected to BHK cells using Lipofectamine (2000) to rescue the virus. Rescued SFV6-2SG-GLuc was aliquoted with cellular debris to allow for improved virus uptake by macrophages in vitro. Wild-type ZIKV from Recife, Brazil, was kindly provided by Alain Kohl at the University of Glasgow. ZIKV was grown in Vero cells and BHK-21 cells, the supernatant was collected then centrifuged to remove cell debris, and virus titers were determined by plaque assays on BHK-21 cells. All viruses used in vivo were passaged once through C6/36 cells. The supernatant from infected C6/36 cells was collected, and infectious virus present in the supernatant was titrated via plaque assay in BHK-21 cells. Viruses were diluted in phosphate-buffered saline with bovine serum albumin (PBSA) to 1 × 10^7^ plaque-forming units (PFU)/mL for injection.

### Mouse Strains.

Wild-type C57BL/6j mice bred in-house at the SBS at the University of Leeds were used in all in vivo experiments unless stated otherwise. Mice were maintained at St. James' Biomedical Services under pathogen-free conditions and used between 4 and 12 wk of age unless stated otherwise. BALB/c mice were purchased from Charles River Laboratories. Mice were age and sex matched in all in vivo experiments. All procedures were carried out in accordance with the United Kingdom Home Office regulations under the authority of the appropriate project and personal license.

### Cell Lines.

Cells were kept at −196 °C for long-term storage. BHK-21 cells were used to grow up virus stock and determining viral titers via plaque assays. BHK-21 cells were cultured at 37 °C with 5% CO_2_ in Glasgow modified essential medium supplemented with 10% tryptose phosphate broth (TPB), 5% fetal calf serum (FCS), and 100 units/mL penicillin and 0.1 mg/mL streptomycin. *Aedes albopictus*-mosquito–derived C6/36 cells were used for growing virus stocks. C6/36 cells were cultured at 28 °C with no added CO_2_ in L-15 media supplemented with 10% TPB, 10% FCS, and 100 units/mL penicillin and 0.1 mg/mL streptomycin. Mouse embryonic fibroblasts (MEFs) from C57BL/6 mice were kept at −196 °C for long-term storage. MEF cells were cultured at 37 °C at 5% CO_2_ in Dulbecco’s modified Eagle medium (DMEM) supplemented with 10% FCS, 100 units/mL penicillin, and 0.1 mg/mL streptomycin and 1% Glutamax in flasks precoated with 0.2% gelatin.

Macrophages were extracted from C57BL/6 mouse bone marrow by flushing cells from the femur using a 26-gauge needle with cold phosphate-buffered saline (PBS). Cells were passed through a 40-μm cell and cultured at 37 °C at 5% CO_2_ in DMEM/F12 supplemented with 10 ng/mL macrophage colony-stimulating factor (M-CSF), 10% FCS, 100 units/mL penicillin and 0.1 mg/mL streptomycin, 1% Glutamax, and 5 mg gentamycin. A total of 4 × 10^5^ cells were seeded in 10 mL media per sterile plastic Petri dish used. At 7 d postextraction, cells were pooled by and adding 3 mL Cellstripper (nonenzymatic cell dissociation solution) to each dish ,and cells were then gently scraped off the plastic. Cells were seeded at a concentration of 2 × 10^5^ per well in a 24-well plate or at 5 × 10^4^ per well in a 96-well plate in complete DMEM/F12.

### Mosquito Rearing and Handling.

The different mosquito species used were *Ae. aegypti* Liverpool strain (a gift of E. Devaney, University of Glasgow, United Kingdom), *Ae. albopictus* La Providence (INFRAVEC2 line; a gift from A.-B. Failloux, Institut Pasteur, France), *Cx pipiens quinquefasciatus* SLAB strain (a gift from M. Weill, Université de Montpellier, France), and *Anopheles coluzzii* Ngousso strain and *An. stephensi* Sda 500 strain (a gift from C. Bourgouin, Institut Pasteur, France). Mosquitoes were reared at 28 °C and 80% humidity conditions with a 12-h light/dark cycle. *Ae. aegypti*, *Ae. albopictus*, and *Cx. pipiens* eggs on filter paper were placed in trays containing ∼1.5 cm of water to hatch overnight. Larvae were fed with Go-cat cat food until pupation. *An. gambiae* and *An. stephensi* eggs were hatched the same day of arrival by placing in water. Ground Tetramin fish flakes were fed to the larvae the first days, following which the larvae were fed with Tetramin pellets until pupation. When pupae formed, these were picked and placed in small water-filled containers and left to emerge into BugDorm mosquito cages. All adult mosquitoes were fed a 10% sucrose solution. Mosquitoes used for salivations and biting experiments were 4 to 8 d old postemergence. Emerging adult mosquitoes were maintained on a 10% (wt/vol) sucrose solution ad libitum. Females were fed with heparinized rabbit blood (Envigo) for 1 h using a 37 °C Hemotek system (Hemotek Ltd).

For salivation of mosquitoes, the mosquito proboscis was placed in a p10 tip containing 0.5 μL immersion oil (Cargille Laboratories). Mosquitoes were then left to salivate for up to an hour before tips were placed in an Eppendorf tube and centrifuged. Saliva droplets were then pooled and stored at −80 °C. Before use, droplets of saliva were carefully pipetted out of the oil under microscope and diluted in PBSA. Saliva from five mosquitoes was utilized per injection unless stated otherwise.

### Antibiotic Treatment.

Mosquitoes were given a 10% sugar solution containing a mixture of 200 units/mL penicillin/streptomycin, gentamycin at 200 μg/mL, and tetracycline at 100 μg/mL, from emergence onward for at least 7 d. Validation of antibiotic treatment was conducted by counting colony-forming units generated following plating on lysogeny broth agar of whole mosquitoes dipped in ethanol to remove any external microbiota (*SI Appendix*, Fig. S2 *E* and *F*).

### In Vivo Mouse Infections.

Mice were anesthetized with 0.1 mL/10 g of Sedator/Ketavet i.p. injection and placed on foil on top of the mosquito cages with the dorsal side of one or both hind feet exposed, allowing a maximum of 5 mosquitoes to feed. Toes were covered with tape to prevent mosquitoes from biting. Mosquitoes were left to feed until fully engorged. Virus injections of either C6/36-derived SFV6 (250 PFU in 1 μL) or SFV4 (10,000 PFU in 1 μL) were made directly at the bite site with a 5-μL 75 N syringe, 33 gauge (Hamilton) using small RN ga33/25-mm needles (Hamilton). Saliva injections were made at a concentration of saliva from five mosquitoes per injection.

Mice were culled via a schedule 1 method. Tissues dissected depended on the experiment but most commonly included skin from a foot and spleen. Blood samples were also collected from the ventricles. Tissue samples collected were stored in 0.5 mL of RNAlater in 1.5-mL tubes, with the exception of spleen and brain samples that were cut in half and stored in 1 mL of RNAlater to enable complete penetration of the RNAlater into the tissue. All samples were left in RNAlater for a minimum of 16 h to prevent RNA degradation. Samples were then stored at 4 °C for short term or at −80 °C for long term. Blood samples were centrifuged and serum was collected and stored at −80 °C until use. Tissue samples were then analyzed via qRT-PCR for an analysis of the expression of SFV virion glycoprotein E1, which is a gene expressed via SG RNA and that has previously been established as a good indicator of total viral RNA levels (genome plus transcripts). Serum was analyzed for viral titers via plaque assays.

### RNA Purification and Quantification.

All tissue samples were lysed in a 1 mL TRIzol reagent and shaken with 7-mm stainless steel beads on a Tissue Lyser at 50 Hz for 10 min to ensure the complete lysis of all tissues. A total of 0.2 mL chloroform was then added to all samples that were then inverted 15 times to allow for gentle mixing of the solutions. Afterward, samples were centrifuged at 12,000g for 15 min at 4 °C in order to separate the mixture into a lower red phenol–chloroform phase and a colorless upper aqueous phase. The upper aqueous phase aqueous phase, containing the RNA, was transferred to a new tube containing an equal amount of 70% ethanol. RNA extractions were performed using the RNA mini purification kit (Life Technologies) by following the protocol provided with the kit. Purified RNA was then stored at −80 °C.

Approximately 1 μg of RNA in a volume of 9 μL of RNase-free water was used for cDNA production using the Applied Biosystems high-capacity RNA-to-cDNA kit. Samples were incubated at 37 °C for 60 min followed by heating to 95 °C for 5 min. The final cDNA was then stored at 4 °C for short-term use and at −20 °C for long-time storage.

Total cDNA was diluted 1 in 5, using RNase-free water, and 1 μL was used per qPCR in 384-well plates. A master mix was made up of primers, water, and SYBR green mix (perfecta, Quantabio.com). A triplicate technical replicate was made for each biological replicate. The generation of a standard curve was accomplished by the dilution of a 10^−2^ PCR-generated standard in a 10-fold serial dilution. A nontemplate control consisting of RNase-free water and the master mix was also included. The PCR plates were run on the Applied Biosystems QuantStudio 7 flex machine.

The cycle threshold (Ct) value was calculated automatically by the QuantStudio software that detects the logarithmic phase of the PCR. Each sample’s relative quantity was calculated based on their position on the standard curve. The standard curve had to have an efficiency close to 100%, which was indicated by the coefficient *R*^2^ ≥ 0.998 and a slope of 3.3. Melt curves were conducted to control for primer specificity. All primer sequences can be found in the *SI Appendix*, Appendix.

### In Vitro Transepithelial Electrical Resistance (TEER) Assay.

Saliva was filter sterilized prior to use in endothelial cell culture. Human umbilical vein endothelial cells (Promocell) were cultured in endothelial growth media (EGM-2; Promocell) at 37 °C in a controlled atmosphere containing 5% CO_2_, until the culture reached 80% confluence. Cells were then detached by trypsinization, and 50,000 cells were seeded on each fibronectin-coated (final concentration, 5 µg/mL in PBS; Merck) Millicell hanging cell culture insert (polyester membrane, pore size of 0.4 µm; Merck Millipore) adapted for 24-well plates. Inserts were cultured in EGM-2, and TEER was measured every day using Millicell electrical resistance system (Merck Millipore) until a plateau was reached, indicating the formation of a complete cell monolayer (normally 3 to 5 d after plating). A total of 50% of the media was replaced with fresh EGM-2 every 2 d. Fresh medium containing SK (1 μM) or mosquito saliva (0.5 eq/mL; 1 µL/mL) was added to both the upper and the lower chamber, and changes in TEER were monitored every 30 min for the first 3 h, then every hour for a total of 8 h. Before each measurement, cells were allowed to reach ambient temperature. Results were normalized against the values of untreated endothelial cells at each time point.

### Tachykinin Peptide Searches.

Searching the InterPro 82.0 database (October 2020) identified matches to the Tachykinin/Neurokinin-like, conserved site (PROSITE: PS00267, F-[IVFY]-G-[LM]-M-[G>]. InterPro: IPR013055) for 1,154 proteins from 219 deuterostomes but only 11 proteins from 7 protostomes. As well as *Ae. aegypti* SK, these included a peptide from a fungus-growing ant (*Trachymyrmex cornetzi*), four peptides from the extremely venomous Brazilian wandering spider (*Phoneutria nigriventer*), four peptides from three species of octopus (Eledoisins from *Eledone cirrhosa*, *Eledone moschata*, and Tachykinins 1 and 2 from *Octopus vulgaris*), and a likely false-positive match to a protein from a tapeworm (*Rodentolepis nana*).

### SK Gene Homology Searches.

Mosquito-focused VectorBase Release 48 (51) and insect-wide OrthoDB v10 (52) gene family resources were searched with the pro-SK precursor protein and corresponding gene (AAEL000229). VectorBase contains annotated genomes of 25 mosquitoes including *Ae. aegypti*, and OrthoDB covers 148 insect species with 56 dipterans of which 17 are mosquitoes including *Ae. aegypti*. Both resources also include two other culicine species, as follows: *Ae. albopictus* and *Culex quinquefasciatus*. Neither resource identified any homologs of AAEL000229 in any of the compared species. Protein sequence searches (BLASTp) of the NCBI nonredundant database returned only AAEL000229 itself (XP_001660125.1) and two other *Ae. aegypti* variants (AAD17916.1 and AAD16885.1). Protein sequence searches (tBLASTn) of all nucleotide data at VectorBase (genomes, transcriptomes, cDNAs, and ESTs) identified AAEL000229 itself in the EST/cDNA and transcript databases, and in the *Ae. aegypti* AaegyL5 genome (Exon1: 5e-04 87%, Exon2 5e-04 100%, Exon3: 1e-29 87.7%) and the genome of the *Ae. aegypti* Aag2 cell line (Exon1: 5e-03 78.3%, Exon2: 5e-03 90%, Exon3: 3e-27 96.4%). No other credible hits were identified; the only hit with a comparable e-value (8e-04) was likely spurious, as it was on the opposite strand of part of the coding region of a much longer gene encoding a DNA polymerase in *Anopheles atroparvus* (AATE013717). The VectorBase region comparison tool, which uses genome-to-genome alignments to identify homologous and orthologous genomic regions between pairs of assemblies, identified no alignable regions in the *Ae. albopictus*, *Culex quinquefasciatus*, or *An. gambiae* genomes. We also searched (tBLASTn) the newly available assembly for *Cx tarsalis* (Main et al., 2020), which returned no significant hits.

### SK Peptide Generation.

SK peptide (N-T-G-D-K-F-Y-G-L-M-amide) was synthesized de novo to >95% purity by Cambridge Research Biochemicals.

### Statistical Analysis.

An analysis of RT-qPCR data was done with Microsoft Excel by the use of the median of the technical replicates and normalizing them to the median of the technical replicates of the housekeeping genes. All data were analyzed with GraphPad Prism software. The nonparametric Kruskal–Wallis test was used for comparisons between more than two groups, while nonparametric Mann–Whitney *U* was used for comparisons between two groups. Ordinary-ANOVA was performed for comparisons between more than two groups of normally distributed data. An analysis of survival curves was conducted using the log-rank (Mantel–Cox) test. All differences were considered significant at *P* < 0.05. All plots have statistical significance indicated as follows: *P < 0.05, **P < 0.01, ***P < 0.001, ****P < 0.0001, and ns = not significant.

Further materials and methods can be found in the *SI Appendix*.

## Supplementary Material

Supplementary File

## Data Availability

All study data are included in the article and/or *SI Appendix*. The data associated with this paper is available from University of Leeds at https://doi.org/10.5518/1163, (56).
