# Supplementary material for: Mosquito saliva enhances virus infection through sialokinin-dependent vascular leakage
Source: Proc Natl Acad Sci U S A. 2022 Jun 8;119(24):e2114309119. doi: 10.1073/pnas.2114309119 (PMC9214539; doi:10.1073/pnas.2114309119)
Supplement: Supplementary File [file pnas.2114309119.sapp.pdf]

Supplementary data

Supplementary Figure 1:

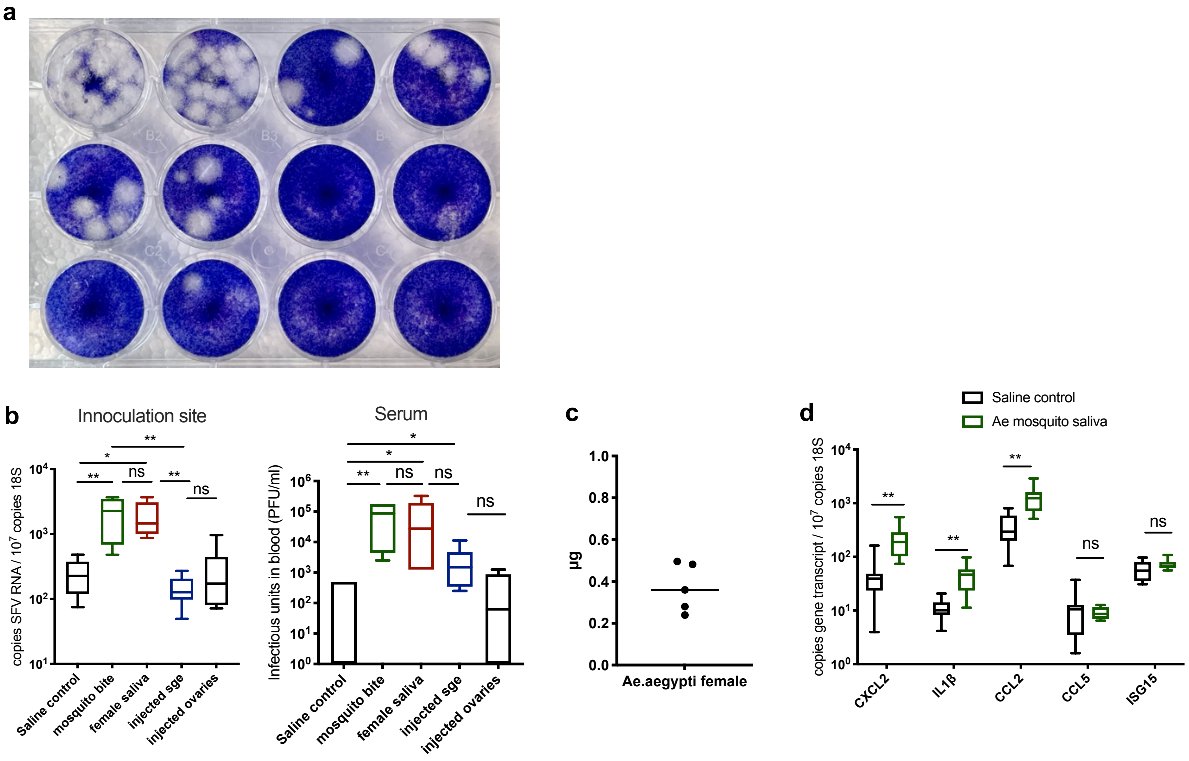

Supplementary Figure 1

(a) Representative SFV plaque assay. Plaques formed in serially diluted samples were quantified by counting.

(b) Mouse skin was inoculated with 10^4 PFU of SFV4 alone or alongside 1.86µg saliva, 5 denatured salivary glands or ovaries, or exposed to up to 5 bites from *Ae. aegypti.*

(c) Saliva acquired from 5 mosquitoes were pooled and protein content was quantified via nanodrop. Each dot represents the average protein concentration per mosquito.

(d) Mouse skin was injected with either saline control or 1.86µg saliva of *Ae. aegypti*. Copy number of host transcripts in the skin was determined by qPCR at 6 hours (n=6)

Supplementary Figure 2:

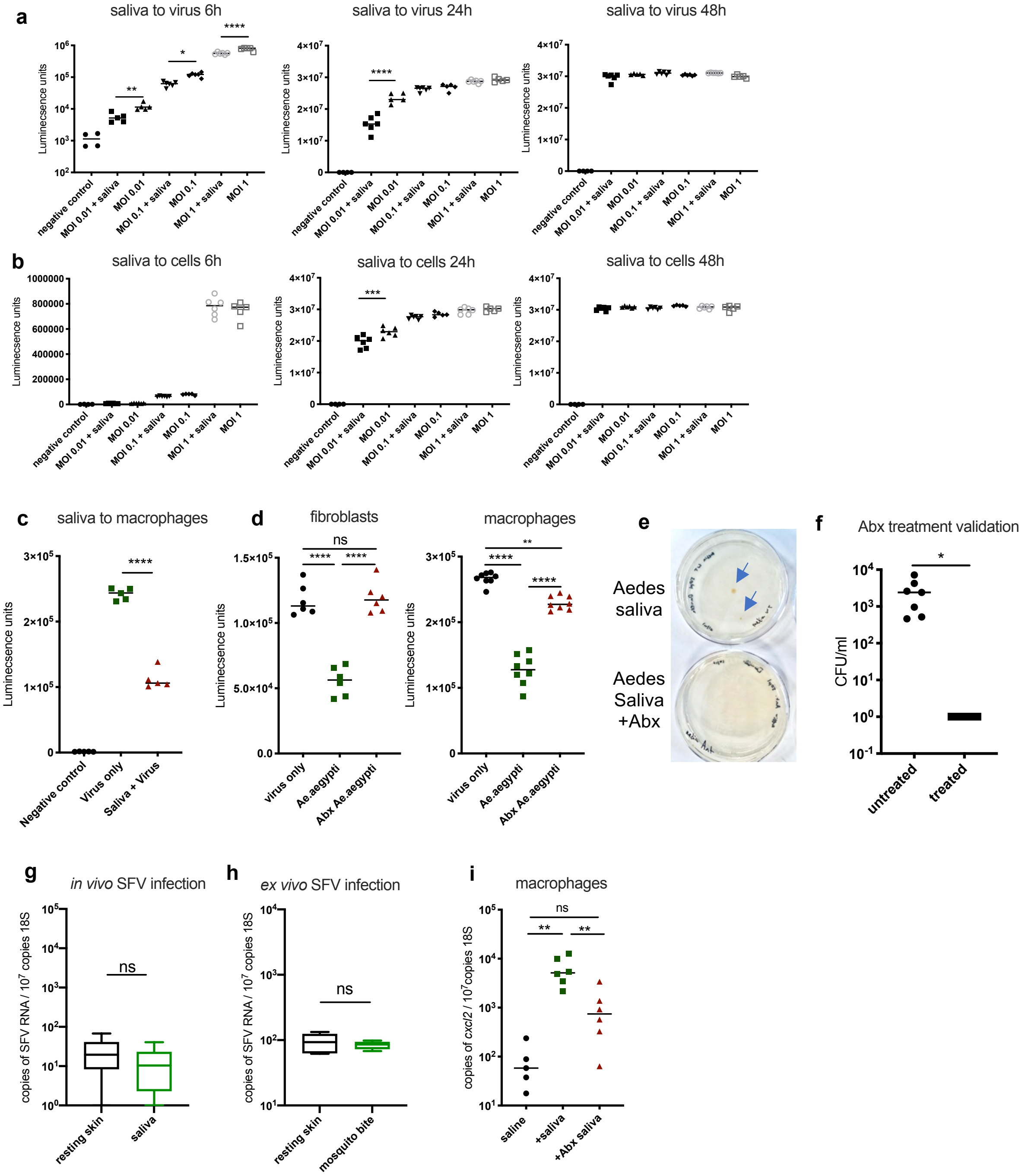

Supplementary Figure 2

(a,b) Primary cultures of dermal fibroblasts (primary target of SFV in skin) where infected with Gluc-expressing SFV at MOIs of 0.01, 0.1 and 1, with or without prior incubation with 2 mosquitoes worth of saliva (0.74 µg protein) and luciferase activity of tissue culture supernatant assayed at 6, 24 and 48 hpi.

(a) Saliva was added to virus (in PBSA) for 20 minutes prior to infection of cells (n=6).

(b) Cells were pre-treated with saliva for 1h in tissue culture media, washed and then infected with SFV (n=6).

(c-d) Primary cultures of macrophages and dermal fibroblasts (both principal targets of SFV in vivo) were infected with Gluc-expressing SFV at an MOI of 0.1 and luciferase activity of tissue culture supernatant assayed at 6 hpi. Cells were exposed to virus with or without 0.67μg of saliva protein per well. Cells were treated with saliva for 1 hour prior to infection at room temperature.
(c) Cells were exposed to virus with or without saliva (n=5).
(d) Cells infected with SFV alone or with saliva from untreated or antibiotic (Abx) treated *Ae. aegypti* mosquitoes (n=8 and n=6).
(e,f) Efficacy validation of antibiotic (Abx) treatment from pupae stage onwards (pen/strep at 200 U per ml, gentamycin at 200 μg/ml, and tetracycline at 100 μg/mL). Mosquitoes at 2 weeks post emergence were dipped in ethanol to remove external microbiota, and saliva obtained as usual (e); or mosquitos dried and whole-body extract plated on agar plates (f) in 10-fold dilutions. CFU/ml was calculated at 24h post plating. Arrows identifying representative colonies for.
(g) Mice were culled and skin immediately infected with 10^5 PFU SFV4 alone or with 1.86μg of mosquito saliva. After 15 minutes, to allow for infection of skin-resident cells, skin was dissected and placed in explant culture for 24h. Viral RNA and host 18S were quantified by qPCR (n=8).
(h) Mouse skin was bitten by Aedes mosquitoes and inflammation allowed to develop for 16 hours, then skin biopsies of this site infected ex vivo with 10^5 PFU SFV and viral RNA and host 18S were quantified by qPCR (n=6).
(i) Macrophages treated with saliva from untreated or Abx-treated *Ae. aegypti* mosquitoes. Expression of *cxcl2* transcripts were measured by qPCR at 6h post treatment (n=6).

Supplementary Figure 3:

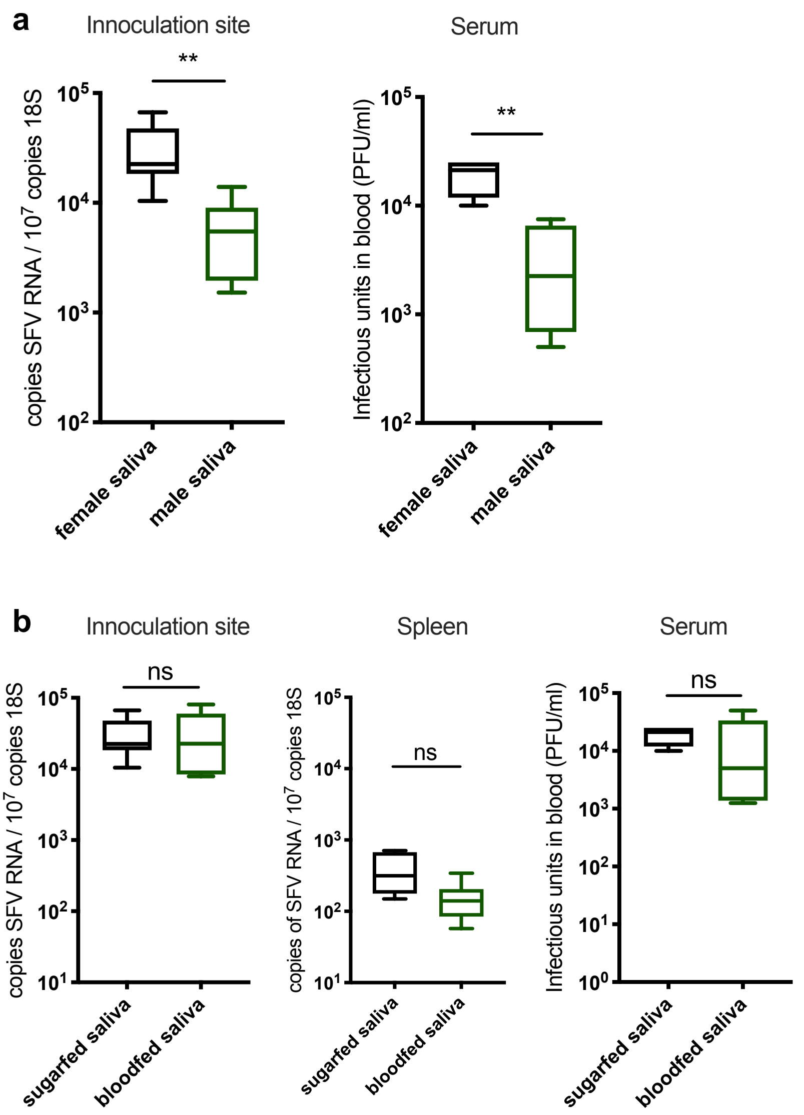

Supplementary Figure 3

(a,b) Mouse skin was inoculated with 10^4 PFU of SFV4 alone or with *Ae. aegypti* saliva in the upper skin of the left foot. Viral RNA and host 18S were quantified from skin and spleen by qPCR and viral titres of serum by plaque assays at 24hpi.

(a) 1 µl male or female *Ae. aegypti* saliva in PBSA, derived from mosquitoes reared in the same cage. Because male saliva contained less total protein, protein content was normalised by diluting female saliva with PBSA prior to injection (n=6).

(b) Saliva from blood fed or exclusively sugar fed female *Ae. aegypti* mosquitoes. (n=6)

Supplementary Figure 4:

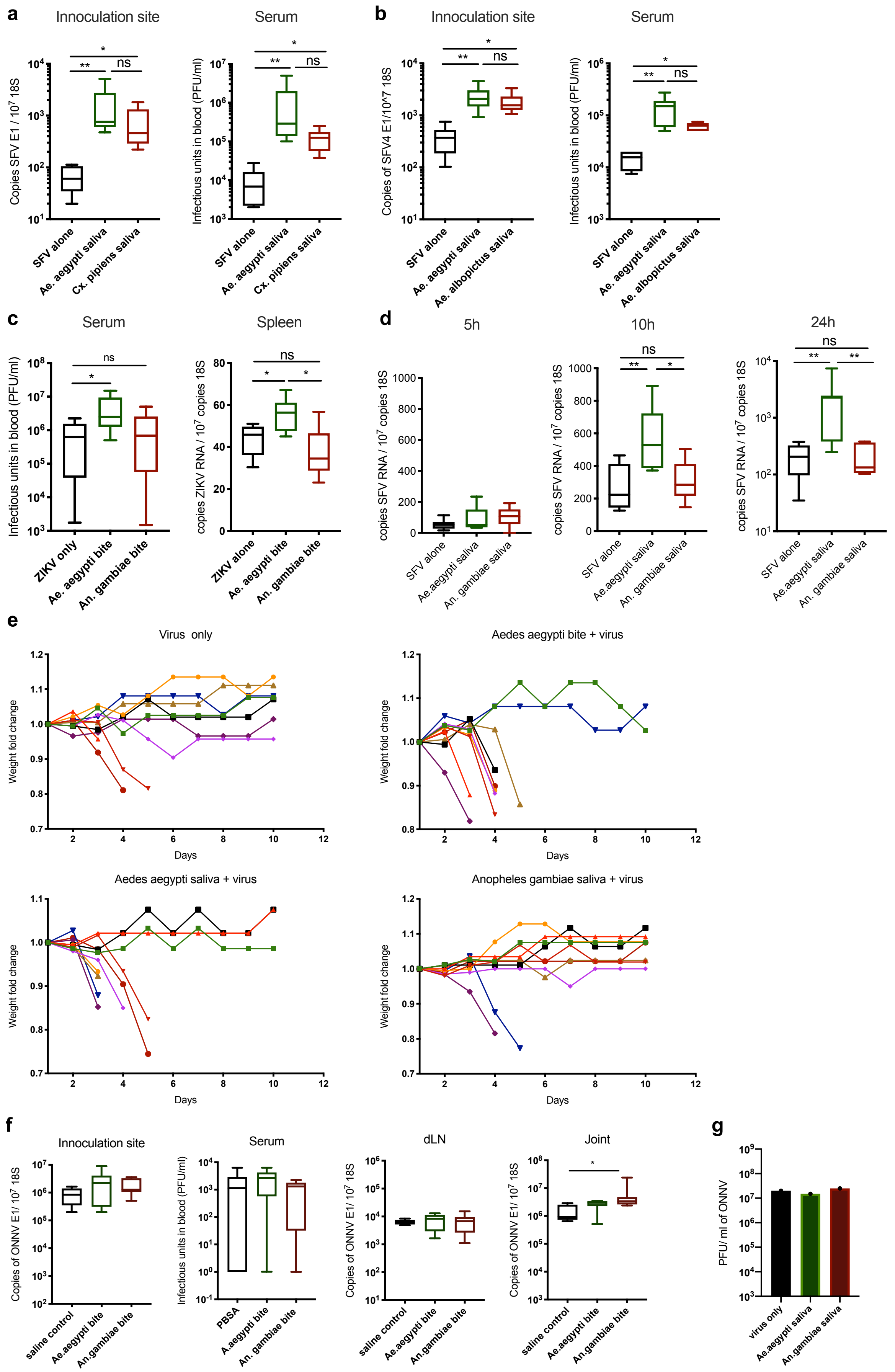

Supplementary Figure 4

(a-e) Mouse skin was inoculated with 10^4 PFU of SFV4 alone or with mosquito saliva from either *Ae. aegypti, Ae. albopictus, Cu. pipiens or An. gambiae* (normalised to protein concentration at 1.86µg / inoculation) . SFV RNA and host 18S and serum viral titres were quantified at 24 hpi.
(c) Mouse skin was exposed to up to 3 bites of either *Ae. aegypti or An. gambiae* mosquitoes.
(d) SFV RNA and host 18S and serum viral titres were quantified at 5, 10 and 24 hpi.
(e) Weights of mice from survival experiment (Fig 4B). Mice were weighed once daily.
(f) Mouse skin was infected with 2 x10^5 PFU ONNV alone or alongside 1.86µg saliva or following up to 3 bites of either *Ae. aegypti* or *An. gambiae* in the upper skin of the left foot. ONNV RNA and host 18S from tissues were quantified by qPCR and serum viral titres were quantified via plaque assays at 48 hpi.
(g) 2 mosquitoes worth of saliva from either *Ae. aegypti* or *An. gambiae, or saline control,* were mixed with multiple tenfold dilution of ONNV, and incubated at 37oC for 60 minutes. The resulting titre was defined by plaque assay on BHK cells. Shown here is a representative ONNV dilution, in this case for 1x10^7 PFU/ml dilution.

Supplementary Figure 5:

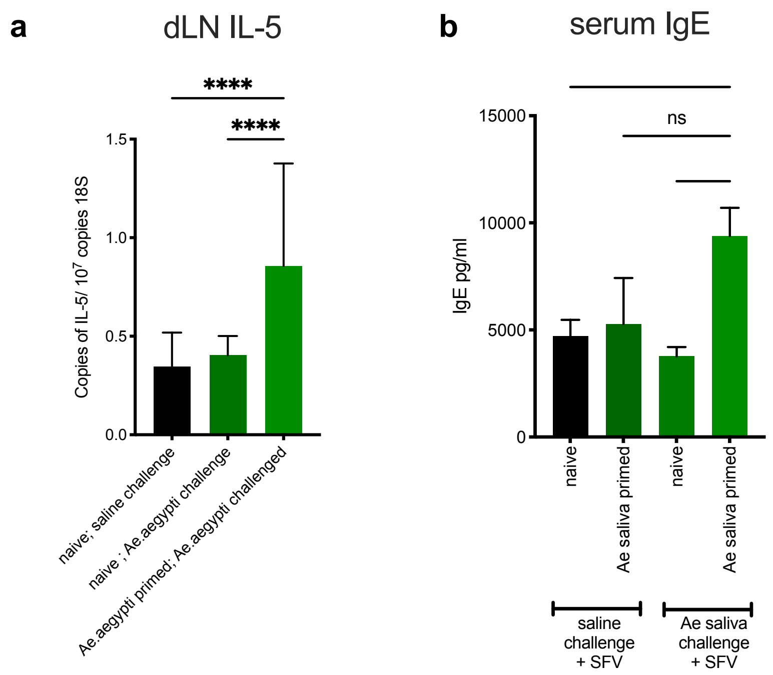

Supplementary Figure 5

(a,b) Balb/c mice skin was inoculated with 10,000 PFU of SFV alone or with *Ae. aegypti* saliva. Mice were either naïve to saliva or primed to saliva by injections of mosquito saliva weekly for 4 consecutive weeks. (A) Draining popliteal lymph node IL-5 transcript expression at 2 hpi were quantified by qPCR (n>6).

(b) Serum total IgE was quantified at 2 hpi by ELISA (n>6).

Supplementary Figure 6:

(a)

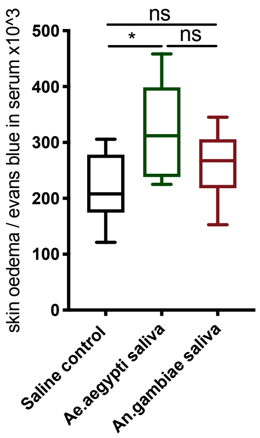

(b)

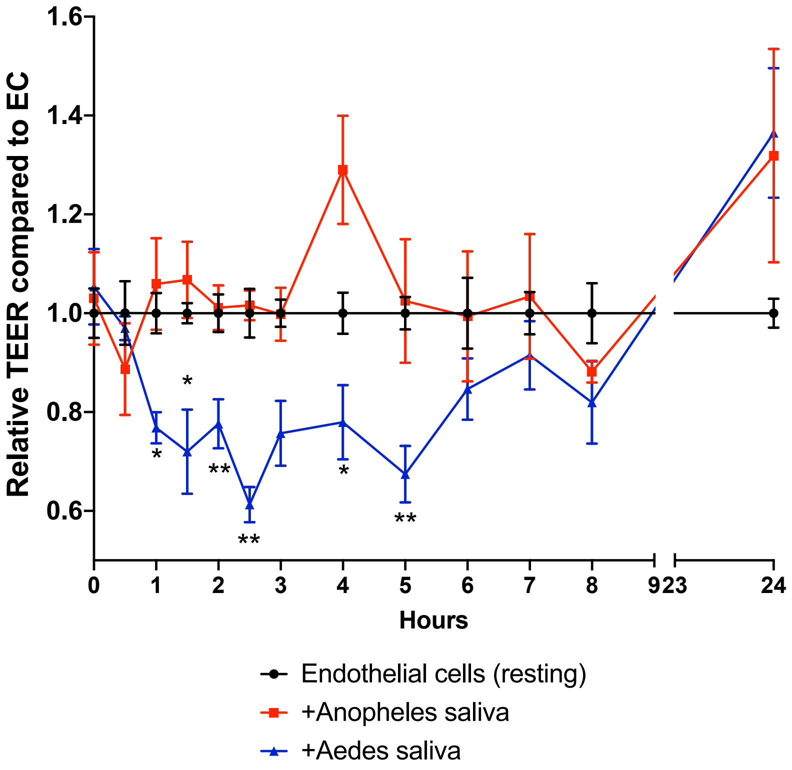

Supplementary Figure 6

(a) Mice administered i.p. with Evans blue, were injected with 1.86µg of mosquito saliva in the skin of naïve or sensitised mice of either *Ae. aegypti or An. gambiae.* Extent of oedema assessed by quantification of Evan's blue dye leakage into skin at 6h post saliva via colorimetric assay (n=6).

(b) Human primary endothelial cell monolayers were treated with either control saline, *Ae. aegypti* or *An. gambiae* and electrical resistance across the monolayer assessed longitudinally. Here, showing the full data set from Figure 5f over a longer time period.

Supplementary Figure 7:

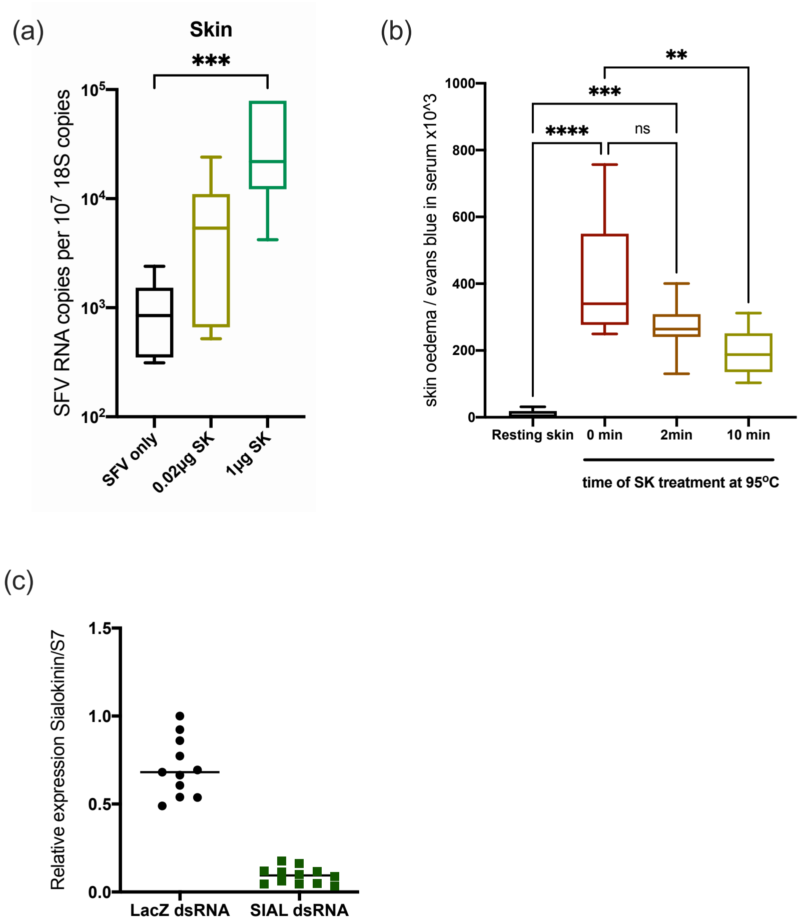

Supplementary Figure 7

(a) Mouse skin was inoculated with 10^4 PFU of SFV4 alone or with sialokinin (total mass used stated) in the upper skin of the left foot. Viral RNA and host 18S were quantified from skin by qPCR at 24hpi. *** Kruskal Wallis-test with Dunn's post-test, p-value=0.0008)

(b) Sialokinin peptide was subject to heat denaturisation for either 0, 2 or 10 minutes at 95°C and 1µg assessed for its ability in induce oedema *in vivo*. Control and heat-treated sialokinin was injected into the skin of mice that had previously received an injection of Evans blue dye (200 µl at 1% w/v s.c. at a distal site, one hour prior to sialokinin administration). 'Resting' mice that had received Evans blue, but no sialokinin, were used as a control. Mice were culled at 30 minutes post sialokinin injection and concentration of Evans blue in skin determined and normalised to levels in the blood. One way ANOVA with Šidák's multiple comparisons test, ns=not significant. ****p<0.0001, ***p<0.001, **p<0.01.

(c) Knockdown efficiency of sialokinin expression in females Ae. aegypti. Expression levels of sialokinin in females previously injected with dsRNA targeting lacZ or sialokinin. Data were analysed using the comparative cycle threshold method using S7 ribosomal protein gene as a standard gene for normalisation. One of the dsLacZ sample was set to RQ=1 and all other samples expressed relatively to this sample. Median plus interquatile range shown. The expression of sialokinin was efficiently knockdown (87% median reduction) in dsSialokinin-injected females compared to dslacZ- ones (Mann-Whitney test, p value <0.0001, n = 11 and 12 pools of 5 females for dsLacZ and dsSialokinin respectively).

Supplementary Appendix

Primers used in this study

Gene Name	Orientation	Sequence	NCBI Reference
18S	Forward	gactcaacacgggaaacctc	NR_003278.1
	Reverse	taaccagacaaatcgctccac	
18S Standard	Forward	cgtagttccgaccataaacga	NR_003278.1
	Reverse	acatctaagggcatcacagacc	
CCL5	Forward	ctgctgctttgcctacctct	NM_013653
	Reverse	acacacttggcggttcctt	
CCL5 Standard	Forward	ccctcaccatcatcctcact	NM_013653
	Reverse	tcagaatcaagaaaccctctatcc	
CXCL10	Forward	tgccacgatgaaaaagaatg	NM_021274
	Reverse	aggggagtgatggagagagg	
CXCL10 Standard	Forward	atccctgcgagcctatcc	NM_021274
	Reverse	aaacttagaactgacgagcctga	
IFN-α	Forward	aggacaggaaggattttgga	NM_010504
	Reverse	gctgctgatggaggtcatt	
IFN-α Standard	Forward	tggctaggctctgtgctttc	NM_010504
	Reverse	ggaggttcctgcatcacac	
IFN-γ	Forward	agcaaggcgaaaaaggatg	NM_008337
	Reverse	ctggacctgtgggttgttg	
IFN-γ Standard	Forward	atctggaggaactggcaaaa	NM_008337
	Reverse	agatacaaccccgcaatcac	
IFN-β	Forward	cacagccctctccatcaact	NM_010510
	Reverse	gcatcttctccgtcatctcc	
IFN-β Standard	Forward	ggcttccatcatgaacaaca	NM_010510
	Reverse	tcccacgtcaatctttcctc	
Rsad2	Forward	tgaagcgtggcggaaagtat	NM_021384.4
	Reverse	tccttcccatctcagcctca	
Rsad2 Standard	Forward	ctgtgcgctggaaggttttc	NM_021384.4
	Reverse	cactggaccttgctcctctg	
IFIT2	Forward	tgcaccacactagcttgca	NM_008331.3
	Reverse	gggatggaagcactcacagt	
IFIT2 Standard	Forward	gcacctctatgtttgagcagtt	NM_008331.3
	Reverse	gcagaaaagtcaaggcaggaa	
ISG15	Forward	cgcagactgtagacacgctta	NM_015783.3

	Reverse	ctcgaagctcagcagaact	
ISG15 Standard	Forward	gtccgtgactaactccatgac	NM_015783.3
	Reverse	tcccaaaagtcctccatacc	
SFV E1	Forward	cgcatcaccttcttttgtg	DQ_189086
	Reverse	ccagaccacccgagatttt	
SFVE1 Standard	Forward	aagtgaagacagcaggtaaggtg	DQ_189086
	Reverse	tatgagttgccccgagtttc	
Zika ENV	Forward	ggaggctgagatggatggt	KX_197192.1
	Reverse	cagtgtttcagccgggatct	
Zika ENV Standard	Forward	aggcaaactgtcgtggttct	KX_197192.1
	Reverse	tcagacccaaccacatcagc	
CXCL2	Forward	aagtttgccttgaccctgaa	NM_009140
	Reverse	tctctttggttcttccgttg	
CXCL2 Standard	Forward	cgcccagacagaagtcatag	NM_009140
	Reverse	actcaccctctccccagaaa	
IL-1**β**	Forward	cgctcagggtcacaagaaac	NM_008361.3
	Reverse	gaggcaaggaggaaaacaca	
IL-1**β** standard	Forward	aaagtatgggctggactgtttc	NM_008361.3
	Reverse	atgtgctggtgcttcattca	
IL-5	Forward	tcctgcctcctcttcctgaa	NM_010558.1
	Reverse	accctgatgcaacgaagagg	
IL-5 standard	Forward	acagagtgggcaatggaagg	NM_010558.1
	Reverse	gggtatgtgatcctcctgcg	
IL-13	Forward	tgccatctacaggacccaga	NM_008355.3
	Reverse	cgtggcgaaacagttgcttt	
IL-13 standard	Forward	gtgtctctccctctgaccct	NM_008355.3
	Reverse	tgagtccacagctgagatgc	
CCL2	Forward	ctcacctgctgctactcattca	NM_011333.3
	Reverse	ccattccttcttggggtca	
CCL2 standard	Forward	caccagcaccagccaact	NM_011333.3
	Reverse	gcatcacagtccgagtcaca	
ONNV E1	Forward	acgctccttccatcacagac	AF192890.1
	Reverse	cggcacctccaaaatcag	
ONNV E1 standard	Forward	gcagtgggcaacataccag	AF192890.1
	Reverse	cggatagtgaccgcatttgt	
S7 (57)	Forward	ccaggctatcctggagttg	XM_001660119.2
	Reverse	gacgtgcttgccggagaac	
Sialokinin (qPCR)	Forward	tgacccttcaacgaaggacg	XM_001660075.2
	Reverse	ttatcaccggtattgagcagg	

Sialokinin (dsRNA synthesis)	Forward	taatacgactcactataggg ttgcagtactatcggaggca	XM_001660075.2
	Reverse	taatacgactcactataggg gcgcactttgtagtatttctcg	

Supplementary Materials and Methods

Luciferase assay. Luciferase assays were performed with SFV6-2SG-GLuc. Bone marrow derived M-CSF macrophages or MEF cells were seeded at a known concentration in 24 or 96 well plates and infected with a known amount of SFV6-2SG-GLuc. Cells were either pre-treated with mosquito saliva, or saliva was added premixed with the virus. For detection of luciferase in macrophages and fibroblasts infected *in vitro* Renilla Luciferase Assay System (Promega) kit was used and samples were run on Mithras LB 940 Multimode Microplate Reader.

Edema measurement.

Edema was measured using systemically injected Evans Blue dye that binds covalently to serum albumin. During normal physiological conditions, the endothelial cell barriers lining the blood vessels prevent the passage of albumin into tissues. When endothelial barrier function is disrupted, however, during inflammation, macromolecules such as albumin can pass through. Therefore, the measuring of the concentration of Evans Blue dye at the site of inflammation was quantified as an indicator of the edema. To determine the amount of fluid accumulation and vascular leakage in the skin, mice were injected subcutaneously (s.c.) with 200 μL of 1% Evans Blue. Skin samples were acquired at 30 min, 3 h, or 6 h post challenge, placed in 250 μL of formamide, and left to soak overnight at 4 °C. Skin samples were then removed from the solution, the dye-stained formamide solution was taken and a 10-fold serial dilution was created by mixing the samples with water. Levels of fluid accumulation were determined using colorimetric measurement of dye concentration at 620 nm using the Multiskan EX. Blood samples were acquired and centrifuged. The amount of dye present in the serum was used as a control for dye present in each mouse. To ensure the complete removal of any residual dye from the blood in the skin tissue, perfusions were carried out immediately after acquiring blood samples. During this process, a 50 mL syringe of PBS with a 26-gauge needle was inserted into the ventricles and the PBS pumped in to ensure the flush out of blood from the entire circulation.

Antihistamine Treatment.

Antihistamine treatment was performed as follows: 0.5 mg cetirizine in 100 μL, 0.02 mg loratadine in 100 μL (approx.1 mg/kg), and 0.1 mg of fexofenadine in 200 μL (approx. 5 mg/kg). Cetirizine and fexofenadine were premixed and given as a 300-μL intraperitoneal (IP) injection, while Loratadine was given as a separate i.p. injection of 100 μL.

Plaque Assays.

BHK-21 cells in 12-well plates were grown to an 80% confluency and infected with virus serial dilutions prepared in 0.75% PBSA (0.75% bovine serum albumin). A total of 200-μL virus dilutions was added to each well and left for 1 h while rocking occasionally. Next, 2× modified Eagle medium supplemented with 4% FCS, 200 units/mL penicillin, and 0.2 mg/mL streptomycin mixed 1:1 with 1.2% Avicel (FMC Biopolymer) was added to the cells. Cells were incubated for 2 d at 37 °C with 5% CO2. Cells were fixed in 10% paraformaldehyde for 1 h and stained with 0.1% Toluidine Blue for at least 30 min. Plaques were counted, and PFU was calculated per millilitre.

ELISA.
An enzyme-linked immunoassay (ELISA) was conducted using Mabtechs ELISA development kit. Plates were read on the Multiskan EX microplate reader (Thermo Scientific) set to 450 nm to measure optical density. Measurements were also taken at 540 nm, and values were subtracted from 450-nm measurements in order to correct for possible optical imperfections in the plate.

Survival.
Mice subjected to neurotropic virus infections were monitored four times daily and weighed every morning for the entire duration of the experiment. Mice demonstrating two or more of predetermined clinical signs were immediately culled. Surviving mice were culled at day 15 post infection.

Mouse Sensitization to saliva.
For sensitization experiments, BALB/c mice were utilized. Mice were subjected to injections of saliva from five mosquitoes in 1 µL of PBSA weekly, for four consecutive weeks. Injections were made on the dorsal side of left hind foot.

Mouse skin explants. Skin was dissected from the hind feet and transferred into 24 well tissue culture plate containing complete DMEM supplemented with 10% FCS, 10% TPB, 5 ml Pen/Strep and 5ml Glutamine broth. Explants were kept at 37°C with 5% CO_2.

dsRNA synthesis and injection into mosquitoes. A 173 bp fragment of the sialokinin coding DNA sequence (AAEL000229, AAEgL5) was amplified from *Ae. aegypti* Liverpool strain cDNAs with KOD Hot Start Master Mix (EMD Millipore) and sialokinin-specific primers with T7 RNA polymerase promoter sequence. The PCR product was purified using the QIAquick Gel Extraction kit (Qiagen). After sequencing, the PCR product was used as a template for a second PCR using the same primers and polymerase. For production of dsLacZ (used as control dsRNA), specific primers with T7 RNA polymerase promoter sequences were used to amplify a lacZ-derived fragment from plasmid template Drosophilaact5C-**β**Gal (Stock number 1220 obtained from DGRC) containing the E. coli lacZ gene. dsRNAs were synthesised and purified using the MEGAscript RNAi kit (Thermo Fisher Scientific) according to the manufacturer's instructions. dsRNA was then purified and concentrated to 10 µg/µl in nuclease free water using 3M Sodium Acetate (Ambion) and ethanol precipitation. At 1 to 2 days after emergence, cold-anesthetised female mosquitoes were injected into their thorax using a nanoinjector (Nanoject II, Drummond Scientific) with 2 µg of dsRNA (dsSialokinin or dsLacZ). Four days post-injection, saliva was collected from dsLacZ- (control) and dsSialokinin-injected females (pools of saliva from 5 females) and after salivation, females (pooled accordingly to saliva pools) were sampled in 1ml of TRIzol (Invitrogen) and stored at -80°C until RNA extraction.

Mosquito RNA extraction and RT-qPCR
Females were homogenized (Precellys 24, Bertin Technologies) in TRIzol (Invitrogen) and samples were centrifuged at 6500g for 30 sec. Total RNA was extracted using the TRIzol method according to the manufacturer's (Invitrogen) protocol except that 1M 1-Bromo-3-ChloroPropane (BCP) (Sigma-Aldrich) was used instead of chloroform. DNase treatment was performed during 30 min at 37°C following the manufacturer's protocol (TURBO DNase, kit

Invitrogen), except that RNasine 0.36 U/µL (Promega) was also added. cDNAs were synthesized using total RNA (25 ng/µL) and M-MLV Reverse Transcriptase (Invitrogen). All cDNAs were aliquoted and stored at -20°C until qPCR. qPCR assays were performed with the Fast SYBR Green Master Mix method (Thermo Fisher Scientific) according to the manufacturer's protocol and using specific primers (Sigma-Aldrich) for genes of interest; reactions were run on an ABI 7500 Fast RT PCR machine and results were analysed with the 7500 Software v2.0.6. To quantify sialokinin knockdown efficiency, data were analysed using the comparative Ct (cycle threshold) method using S7 ribosomal protein gene as a standard gene for normalisation. One of the dsLacZ sample was set to a relative quantification (RQ) of 1 and all other samples expressed relatively to this sample.
